# CB2 Receptor Activation Attenuates IL-1β-Induced Inflammatory Transcriptomic Networks in Human Gingival Fibroblasts

**DOI:** 10.3390/ph19091406

**Published:** 2026-09-06

**Authors:** Uswa Arain, Keegan Dedman, Matthew Cooper, Obaed Ashfaq, Karima Ait-Aissa, Undral Munkhsaikhan, Ehsanul Hoque Apu, Amal Majed Sahyoun, Mustafa Dabbous, Modar Kassan, Ammaar H. Abidi

**Affiliations:** 1 College of Dental Medicine, Lincoln Memorial University, Knoxville, TN 37917, USA; uswa.arain@lmunet.edu (U.A.); matthew.cooper@lmunet.edu (M.C.); obaed.ashfaq@lmunet.edu (O.A.); undral.munkhsaikhan@lmunet.edu (U.M.); ehoqueapu@neomed.edu (E.H.A.); amal.sahyoun@lmunet.edu (A.M.S.); 2 Department of Biomedical Sciences, Bitonte College of Dentistry, Northeast Ohio Medical University, Rootstown, OH 44272, USA; 3 College of Dentistry, The University of Tennessee Health Science Center, Memphis, TN 38163, USA

**Keywords:** CB2 receptor, HU-308, periodontitis, human gingival fibroblasts, IL-1β, inflammation, transcriptomics, extracellular matrix, nitric oxide, glucose transporters, G protein-coupled receptors

## Abstract

**Background**: Periodontitis is a chronic inflammatory disease characterized by dysregulated host immune responses that drive connective tissue destruction and alveolar bone loss. Human gingival fibroblasts (HGFs) are central regulators of periodontal inflammation through their production of cytokines, chemokines, matrix-remodeling enzymes, and other inflammatory mediators. Although cannabinoid receptor 2 (CB2) activation has demonstrated anti-inflammatory properties, its coordinated effects on multiple inflammatory pathways in gingival fibroblasts remain poorly understood. This study investigated the transcriptomic effects of the selective CB2 agonist HU-308 on IL-1β-induced inflammatory responses in HGFs. **Methods**: Primary HGFs were divided into untreated controls, IL-1β-stimulated cells (10 ng/mL), and IL-1β-stimulated cells treated with HU-308 (10 μM). Twenty-four hours after stimulation, transcriptome-wide expression profiling was performed using Affymetrix Human Clariom S microarrays, followed by targeted analysis of selected inflammation-related transcriptional domains. Selected transcripts were evaluated within predefined biological domains including cytokines, chemokines, extracellular matrix-associated genes, NO/cGMP-related genes, transporter-associated genes, and GPCR-related transcripts. Gene expression was analyzed using one-way ANOVA with Tukey’s post hoc test. **Results**: IL-1β induced a coordinated inflammatory transcriptional program characterized by increased expression of pro-inflammatory cytokines, chemokines, matrix metalloproteinases, glucose transporter genes, and multiple GPCR-related transcripts, while suppressing collagen-associated genes, NOS3, GPR4, and GPR78. HU-308 broadly attenuated these inflammatory responses by reducing the expression of cytokines, chemokines, matrix metalloproteinases, and several GPCR-related genes while restoring collagen-associated transcripts, nitric oxide signaling components, anti-inflammatory mediators, and selected glucose transporters toward basal levels. Schematic multidimensional visualizations were used to illustrate relative expression patterns among selected transcripts within each functional domain; these visualizations do not represent statistically derived gene networks or molecular interactions. **Conclusions**: Pharmacological modulation of CB2 by HU-308 exerts broad immunomodulatory effects in IL-1β-stimulated human gingival fibroblasts by coordinately regulating multiple transcriptional networks involved in periodontal inflammation. These findings demonstrate that HU-308 treatment is associated with coordinated modulation of inflammatory, extracellular matrix, nitric oxide, metabolic, and GPCR-associated transcriptional pathways in IL-1β-stimulated HGFs. The results support the hypothesis that CB2 signaling may participate in the broader regulation of these interconnected responses; however, receptor-specific studies using CB2 antagonism or CNR2 knockdown are required to establish causality.

## 1. Introduction

Periodontal disease (PD) is a highly prevalent chronic inflammatory disorder affecting approximately 743 million people worldwide and represents a major public health burden because of its detrimental effects on oral function, aesthetics, quality of life, and systemic health [1]. Although initiated by dysbiotic bacterial biofilms, periodontal tissue destruction is primarily driven by a dysregulated host immune response characterized by sustained production of inflammatory cytokines and chemokines, excessive immune-cell recruitment, extracellular matrix degradation, oxidative stress, and metabolic reprogramming, which collectively promote connective tissue breakdown and alveolar bone loss [2,3,4]. These observations have shifted the current understanding of periodontitis from a purely bacteria-driven disease to one in which host-mediated inflammatory mechanisms play a central role in disease progression.

Among the numerous inflammatory mediators implicated in periodontitis, interleukin-1β (IL-1β) is recognized as a principal driver of periodontal inflammation [5]. IL-1β functions as a master pro-inflammatory cytokine by activating multiple downstream signaling cascades that stimulate the production of additional cytokines, chemokines, and matrix metalloproteinases (MMPs), while simultaneously influencing nitric oxide signaling, oxidative stress, and cellular metabolism [6,7]. Through these coordinated actions, IL-1β promotes immune-cell recruitment, extracellular matrix degradation, impaired tissue repair, and persistent inflammatory activation within periodontal tissues [5,8]. Although many studies have investigated individual inflammatory mediators or isolated signaling pathways, considerably less is known about how these diverse biological processes interact within a unified inflammatory network during periodontal disease.

Human gingival fibroblasts (HGFs) are among the principal resident stromal cells within the gingival connective tissue and play a central role in maintaining periodontal homeostasis [9,10]. Beyond their structural function, HGFs actively participate in innate immune responses by producing cytokines, chemokines, extracellular matrix proteins, matrix-degrading enzymes, and other inflammatory mediators that regulate leukocyte recruitment, connective tissue remodeling, wound healing, and tissue repair [10,11]. Consequently, HGFs provide a biologically relevant in vitro model for investigating how inflammatory stimulation coordinately alters multiple interconnected signaling pathways involved in periodontal disease progression [12].

In recent years, the endocannabinoid system (ECS) has emerged as an important regulator of immune and inflammatory responses [13,14]. Cannabinoid signaling influences numerous cellular processes, including immune-cell activation, oxidative stress, apoptosis, autophagy, and tissue remodeling, and has therefore attracted increasing attention as a potential therapeutic target in oral inflammatory diseases [13,14]. Among cannabinoid receptors, cannabinoid receptor type 2 (CB2) is a Gi/o protein-coupled receptor (GPCR) predominantly expressed in immune and peripheral tissues and functions as a critical negative regulator of inflammation. Activation of CB2 inhibits adenylyl cyclase, reduces intracellular cyclic AMP (cAMP) production, and suppresses multiple pro-inflammatory signaling pathways, including nuclear factor kappa B (NF-κB) and mitogen-activated protein kinase (MAPK) signaling [15]. Increasing evidence also suggests that CB2 signaling may involve β-arrestin-dependent pathway bias, allowing selective regulation of inflammatory responses rather than generalized immunosuppression. Collectively, these properties identify CB2 as a promising target for host-modulatory therapy in periodontal disease [16].

Beyond CB2 itself, inflammation is increasingly recognized as influencing the expression of numerous G protein-coupled receptors (GPCRs), many of which regulate cellular responses to inflammatory mediators, chemokines, metabolites, extracellular pH, and tissue injury [17,18]. Because GPCRs represent the largest family of membrane receptors involved in inflammatory signaling, alterations in GPCR-associated transcription may substantially influence fibroblast behavior during chronic inflammation [19]. However, little is currently known regarding how inflammatory activation remodels GPCR-associated transcriptional networks in human gingival fibroblasts or whether selective CB2 modulation influences these broader receptors signaling programs.

Despite growing interest in CB2-mediated immunomodulation, its integrated effects on the complex molecular networks underlying periodontal inflammation remain poorly understood. Periodontitis is driven by the coordinated interaction of inflammatory cytokines, chemokines, extracellular matrix remodeling, oxidative stress, metabolic adaptation, and receptor-mediated signaling rather than by isolated molecular pathways [20]. To date, no study has comprehensively examined how selective CB2 activation simultaneously regulates these interconnected biological processes within the same IL-1β-stimulated gingival fibroblast model.

Therefore, the present study employed transcriptomic profiling of IL-1β-stimulated human gingival fibroblasts to investigate the effects of the selective CB2 receptor agonist HU-308 across multiple disease-relevant biological domains. Specifically, we examined coordinated changes in interleukin signaling, chemokine networks, extracellular matrix remodeling, nitric oxide/cGMP signaling, glucose transporter-associated metabolic pathways, and GPCR-related transcriptional networks. By integrating these pathways within a single experimental model, this systems-level transcriptomic analysis was designed to determine whether pharmacological treatment with the selective CB2 agonist HU-308 is associated with coordinated modulation of multiple IL-1β-responsive transcriptional networks rather than changes in isolated inflammatory mediators. The resulting data were also used to develop a hypothesis regarding a broader regulatory role for CB2 signaling that can be tested in future receptor-specific mechanistic studies.

## 2. Results

### 2.1. HU-308 Attenuates IL-1β-Induced Interleukin Expression in Human Gingival Fibroblasts (HGFs)

**Figure 1:**
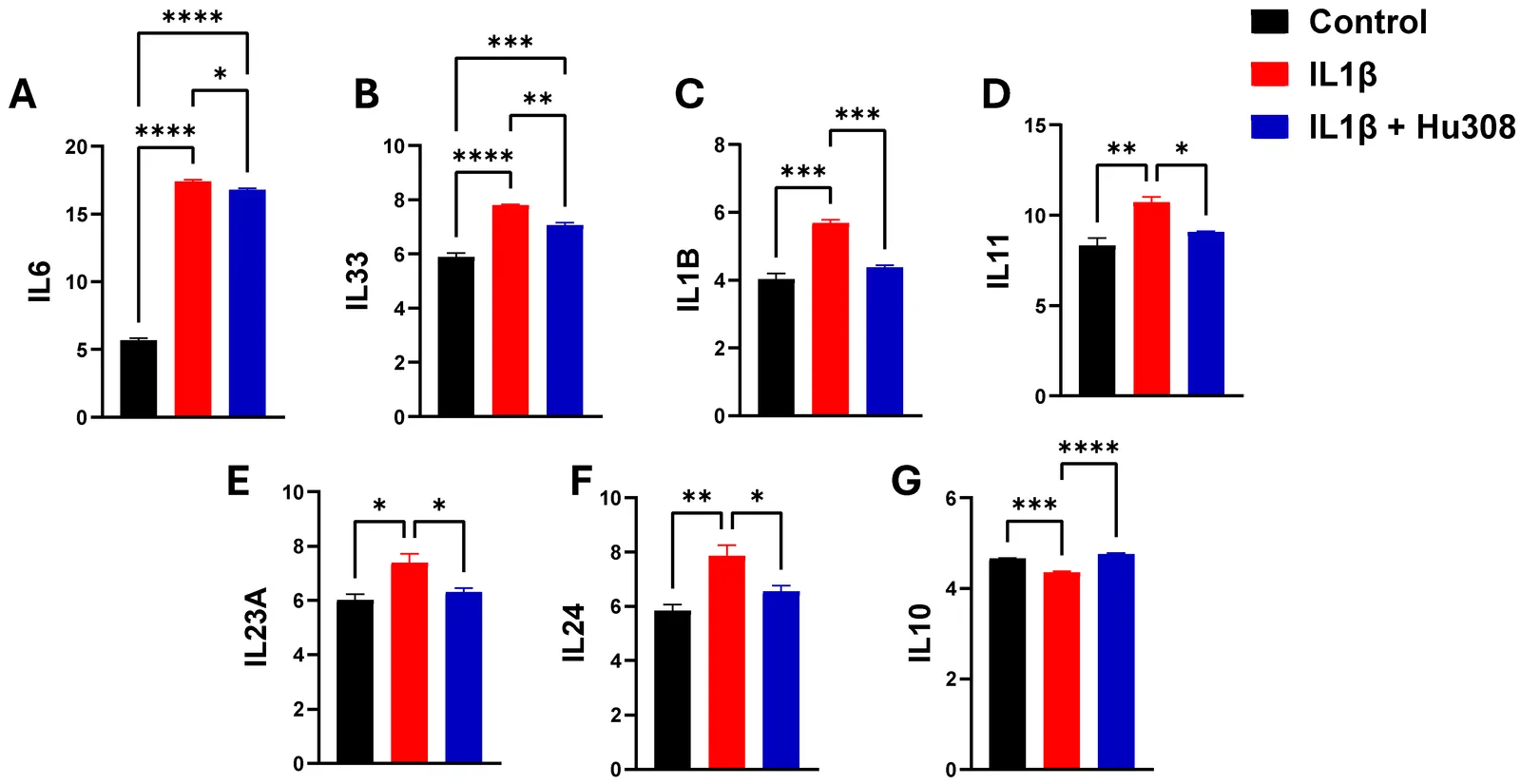
**HU-308 attenuates IL-1β-induced interleukin expression in human gingival fibroblasts.** Human gingival fibroblasts (HGFs) were divided into three experimental groups: untreated Control (black bars), IL-1β (10 ng/mL)-stimulated cells (red bars), and cells treated with IL-1β followed by the selective CB2 receptor agonist HU-308 (10 μM; blue bars). Twenty-four hours after IL-1β stimulation, transcript levels of proinflammatory and anti-inflammatory interleukins were quantified. IL-1β stimulation significantly increased the expression of the pro-inflammatory cytokines IL1B, IL6, IL23A, IL33, IL11, IL1α, and IL24, and treatment with HU-308 attenuated the IL-1β-induced expression of these cytokines, indicating suppression of the inflammatory response (**A**–**F**). In contrast, IL10 exhibited reduced expression following IL-1β stimulation, and HU-308 treatment partially restored its expression, supporting a shift toward a less inflammatory phenotype following CB2 receptor activation (**G**). Data are presented as mean ± SEM from three independent biological replicates (*n* = 3 per group). Statistical significance was determined by one-way ANOVA followed by Tukey’s multiple-comparison test. * *p* < 0.05, ** *p* < 0.01, *** *p* < 0.001, and **** *p* < 0.0001 was considered statistically significant.

To determine whether CB2 receptor activation modulates inflammatory cytokine expression, transcript levels of pro-inflammatory and anti-inflammatory interleukins were quantified in untreated HGFs, IL-1β-stimulated cells, and IL-1β-stimulated cells treated with the selective CB2 agonist HU-308 (Figure 1).

IL-1β stimulation elicited a robust inflammatory transcriptional response characterized by significant upregulation of multiple pro-inflammatory interleukins. Among these, IL6 exhibited one of the largest responses, increasing markedly following IL-1β stimulation compared with untreated controls (**** *p* < 0.0001; Figure 1A). Although HU-308 significantly reduced IL6 expression relative to the IL-1β group (* *p* < 0.05; Figure 1A), transcript levels remained significantly higher than those of untreated cells (**** *p* < 0.0001; Figure 1A), indicating partial suppression of the IL-1β-induced response.

A similar pattern was observed for IL33, which was induced following IL-1β stimulation compared with untreated controls (**** *p* < 0.0001; Figure 1B). HU-308 significantly attenuated IL33 expression relative to the IL-1β group (** *p* < 0.01; Figure 1B); however, transcript levels remained significantly elevated compared with control cells (*** *p* < 0.001; Figure 1B).

Expression of IL1 B was also significantly increased following IL-1β stimulation (*** *p* < 0.001 versus control; Figure 1C). Treatment with HU-308 markedly reduced IL1 β expression (*** *p* < 0.001 versus IL-1B; Figure 1C), restoring transcript levels to those of untreated controls, with no significant difference observed between the HU-308 and control groups.

Likewise, IL11 expression increased significantly following inflammatory stimulation (** *p* < 0.01 versus control; Figure 1D). HU-308 significantly reduced IL11 expression relative to IL-1β-treated cells (* *p* < 0.05; Figure 1D), completely reversing the IL-1β-induced increase such that expression was no longer significantly different from untreated controls.

The cytokine IL23A was likewise significantly induced following IL-1β exposure (* *p* < 0.05 versus control; Figure 1E). Activation of CB2 receptors significantly decreased IL23A expression compared with IL-1β-treated cells (* *p* < 0.05; Figure 1E), restoring transcript levels to values comparable with untreated controls.

Similarly, IL24 expression was significantly increased following IL-1β stimulation (** *p* < 0.01 versus control; Figure 1F). HU-308 significantly attenuated this induction (* *p* < 0.05 versus IL-1β; Figure 1F), returning IL24 expression to a level that was no longer significantly different from that of the control group.

In contrast to the pro-inflammatory cytokines, the anti-inflammatory mediator IL10 exhibited the opposite pattern of regulation. IL10 expression was significantly suppressed following IL-1β stimulation (*** *p* < 0.001 versus control; Figure 1G). HU-308 significantly increased IL10 expression relative to IL-1β-treated cells (**** *p* < 0.0001; Figure 1G), restoring expression to baseline levels such that no significant difference remained between the HU-308-treated and control groups.

Collectively, these findings demonstrate that IL-1β induces a broad pro-inflammatory cytokine program in HGFs. Activation of CB2 receptors by HU-308 significantly attenuated IL-1β-induced cytokine expression, completely restoring IL1B, IL11, IL23A, IL24, IL10, and IL15 expression to levels comparable with untreated controls, while partially reducing the induction of IL6 and IL33. These results indicate that HU-308 treatment effectively reverses most IL-1β-driven inflammatory transcriptional changes in HGFs.

### 2.2. HU-308 Suppresses IL-1β-Induced Chemokine Expression in Human Gingival Fibroblasts

**Figure 2:**
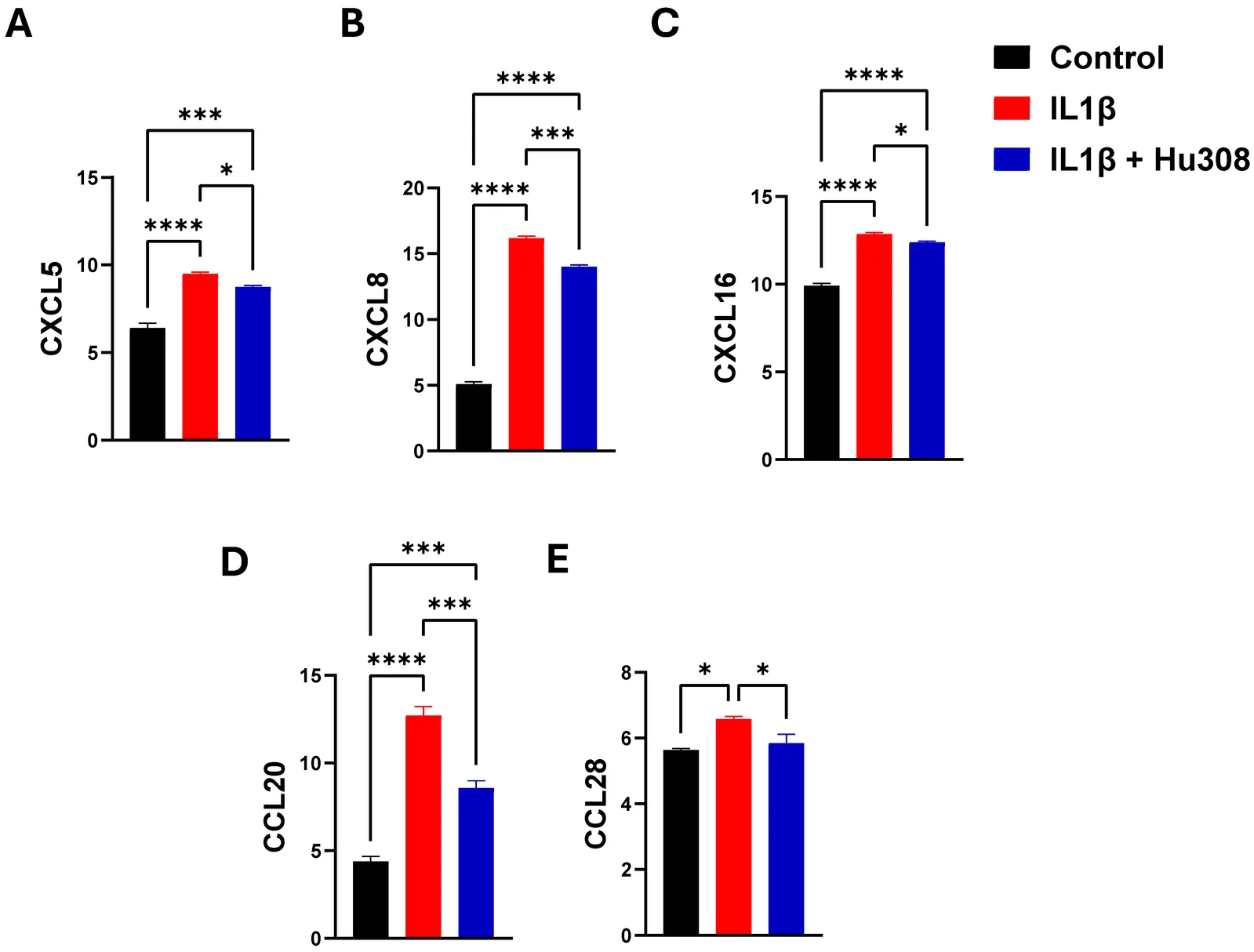
**HU-308 modulates IL-1β-induced chemokine expression in human gingival fibroblasts.** Human gingival fibroblasts (HGFs) were divided into three experimental groups: untreated Control (black bars), IL-1β (10 ng/mL)-stimulated cells (red bars), and cells treated with IL-1β followed by the selective CB2 receptor agonist HU-308 (10 μM; blue bars). Twenty-four hours after IL-1β stimulation, transcript levels of inflammatory chemokines were quantified. IL-1β stimulation significantly increased the expression of CXCL5, CXCL8, CXCL16, CCL20, and CCL28, consistent with activation of chemokine-mediated inflammatory signaling (**A**–**E**). Treatment with HU-308 attenuated the IL-1β-induced expression of these chemokines, indicating suppression of inflammatory chemokine responses and reduced potential for immune-cell recruitment (**A**–**E**). Data are presented as mean ± SEM from three independent biological replicates (*n* = 3 per group). Statistical significance was determined by one-way ANOVA followed by Tukey’s multiple-comparison test. * *p* < 0.05, *** *p* < 0.001, and **** *p* < 0.0001 was considered statistically significant.

To determine whether CB2 receptor activation modulates chemokine production during inflammation, transcript levels of CXCL5, CXCL8, CXCL16, CCL20, and CCL28 were quantified in untreated HGFs, IL-1β-stimulated cells, and IL-1β-stimulated cells treated with the selective CB2 receptor agonist HU-308 (Figure 2).

IL-1β stimulation led to a pronounced increase in the expression of all chemokines examined, indicating activation of inflammatory chemokine signaling in HGFs. CXCL5 expression increased significantly following IL-1β stimulation compared with untreated controls (**** *p* < 0.0001; Figure 2A). Treatment with HU-308 significantly reduced CXCL5 expression relative to the IL-1β group (* *p* < 0.05; Figure 2A); however, transcript levels remained significantly higher than those observed in untreated controls (*** *p* < 0.001; Figure 2A), indicating partial attenuation of the inflammatory response.

A similar expression profile was observed for CXCL8, which exhibited robust induction following IL-1β stimulation (**** *p* < 0.0001 versus control; Figure 2B). HU-308 significantly suppressed CXCL8 expression compared with IL-1β-treated cells (*** *p* < 0.001**;** Figure 2B), although expression remained significantly elevated above basal levels (**** *p* < 0.0001 versus control; Figure 2B).

Among the CXC chemokines, CXCL16 demonstrated marked upregulation following IL-1β stimulation (**** *p* < 0.0001 versus control; Figure 2C). Activation of CB2 receptors significantly reduced CXCL16 transcript levels compared with the IL-1β group (* *p* < 0.05; Figure 2C); however, expression remained significantly greater than that of untreated cells (**** *p* < 0.0001; Figure 2C).

Likewise, CCL20 was strongly induced by IL-1β treatment (**** *p* < 0.0001 versus control; Figure 2D). HU-308 markedly attenuated this response (*** *p* < 0.001 versus IL-1β; Figure 2D), although CCL20 expression remained significantly elevated relative to control cells (*** *p* < 0.001; Figure 2D), indicating partial normalization of inflammatory chemokine expression.

In contrast, CCL28 exhibited a more modest response to inflammatory stimulation. IL-1β significantly increased CCL28 expression compared with untreated controls (* *p* < 0.05; Figure 2E), and HU-308 significantly reduced transcript abundance relative to IL-1β-treated cells (* *p* < 0.05; Figure 2E). Following HU-308 treatment, CCL28 expression was restored to a level that was no longer significantly different from untreated controls.

Collectively, these findings demonstrate that IL-1β induces a broad chemokine transcriptional program in HGFs, whereas HU-308-mediated CB2 receptor activation significantly suppresses the expression of multiple inflammatory chemokines. While the IL-1β-induced increases in CXCL5, CXCL8, CXCL16, and CCL20 were only partially attenuated, HU-308 restored CCL28 expression to baseline levels, indicating differential regulation of individual chemokines by CB2 signaling.

### 2.3. HU-308 Attenuates IL-1β-Induced Matrix Metalloproteinase Expression in Human Gingival Fibroblasts

**Figure 3:**
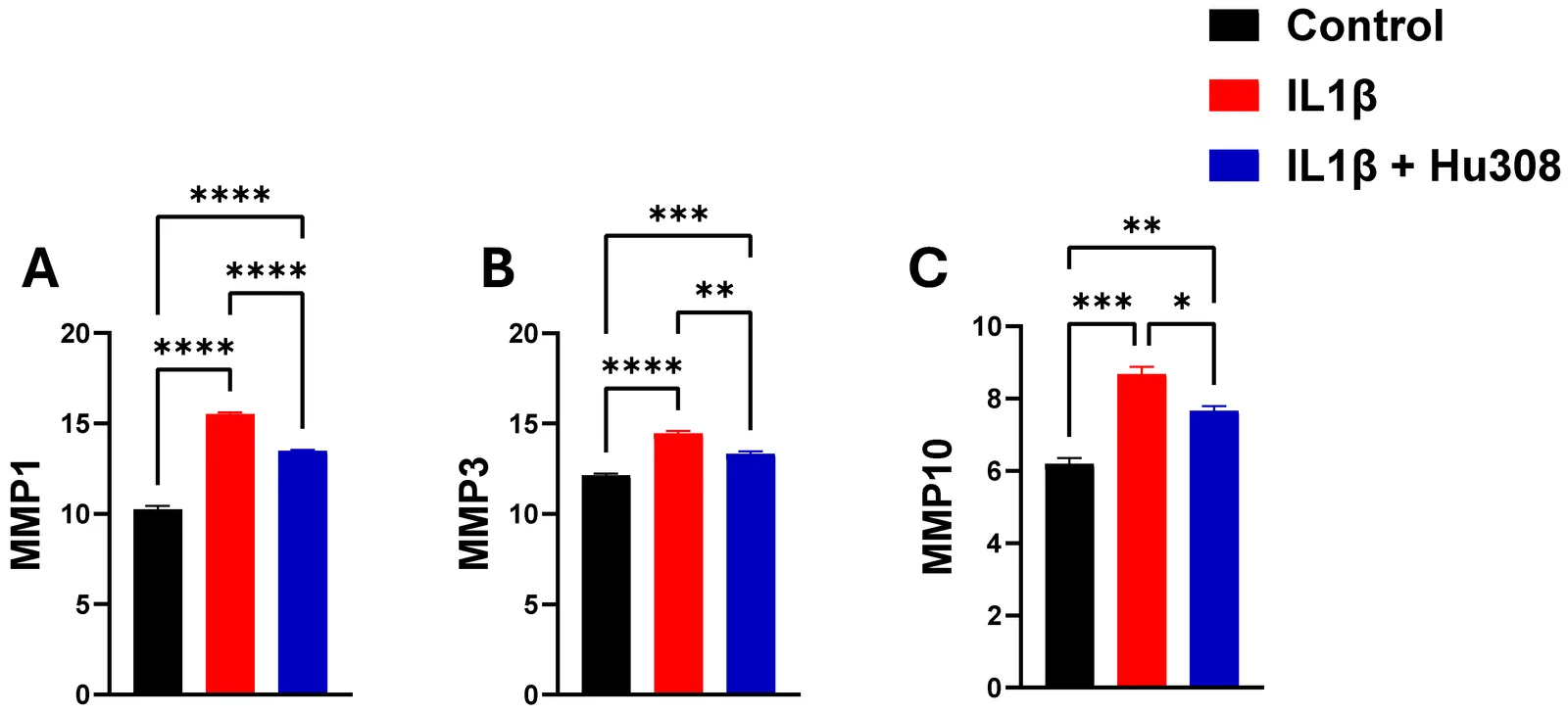
**HU-308 attenuates IL-1β-induced matrix metalloproteinase expression in human gingival fibroblasts.** Human gingival fibroblasts (HGFs) were divided into three experimental groups: untreated Control (black bars), IL-1β (10 ng/mL)-stimulated cells (red bars), and cells treated with IL-1β followed by the selective CB2 receptor agonist HU-308 (10 μM; blue bars). Twenty-four hours after IL-1β stimulation, transcript levels of MMP1, MMP3, and MMP10 were quantified. IL-1β stimulation significantly increased the expression of all three matrix metalloproteinases, consistent with enhanced extracellular matrix degradation and tissue remodeling associated with periodontal inflammation (**A**–**C**). Treatment with HU-308 significantly attenuated the IL-1β-induced expression of MMP1, MMP3, and MMP10, suggesting that CB2 receptor modulation suppresses matrix-degrading pathways and may contribute to the preservation of periodontal tissue integrity (**A**–**C**). Data are presented as mean ± SEM from three independent biological replicates (*n* = 3 per group). Statistical significance was determined by one-way ANOVA followed by Tukey’s multiple-comparison test. * *p* < 0.05, ** *p* < 0.01, *** *p* < 0.001, and **** *p* < 0.0001 was considered statistically significant.

To investigate whether CB2 receptor activation influences extracellular matrix remodeling during inflammation, transcript levels of the matrix metalloproteinases MMP1, MMP3, and MMP10 were quantified in untreated HGFs, IL-1β-stimulated cells, and IL-1β-stimulated cells treated with the selective CB2 receptor agonist HU-308 (Figure 3).

IL-1β stimulation significantly increased the expression of all three matrix metalloproteinases compared with untreated controls, indicating activation of extracellular matrix degradation-associated genes. MMP1 expression was markedly elevated following IL-1β stimulation compared with untreated controls (**** *p* < 0.0001; Figure 3A). Treatment with HU-308 significantly reduced MMP1 transcript levels relative to the IL-1β group (**** *p* < 0.0001; Figure 3A), although expression remained significantly higher than that observed in untreated control cells (**** *p* < 0.0001; Figure 3A), indicating partial attenuation of the IL-1β-induced response.

A similar expression pattern was observed for MMP3, which was robustly induced following IL-1β stimulation (**** *p* < 0.0001 versus control; Figure 3B). Activation of CB2 receptors significantly suppressed MMP3 expression compared with IL-1β-treated cells (** *p* < 0.01; Figure 3B). However, MMP3 transcript levels remained significantly elevated relative to untreated controls (*** *p* < 0.001; Figure 3B), demonstrating partial normalization rather than complete restoration.

Likewise, MMP10 exhibited the greatest induction among the metalloproteinases analyzed following IL-1β exposure (*p* < 0.0001 versus control; Figure 3C). HU-308 significantly attenuated this increase (* *p* < 0.05 versus IL-1β; Figure 3C), although MMP10 expression remained significantly above basal levels (** *p* < 0.001 versus control; Figure 3C), indicating that HU-308 treatment reduced, but did not completely reverse, the IL-1β-mediated induction of MMP10.

Collectively, these findings demonstrate that IL-1β strongly activates matrix metalloproteinase gene expression in HGFs, whereas pharmacological activation of CB2 receptors significantly suppresses the expression of MMP1, MMP3, and MMP10. Although HU-308 did not completely restore metalloproteinase expression to basal levels, it consistently attenuated the IL-1β-induced transcriptional activation of genes involved in extracellular matrix degradation.

### 2.4. HU-308 Modulates IL-1β-Suppressed Collagen Gene Expression in Human Gingival Fibroblasts

**Figure 4:**
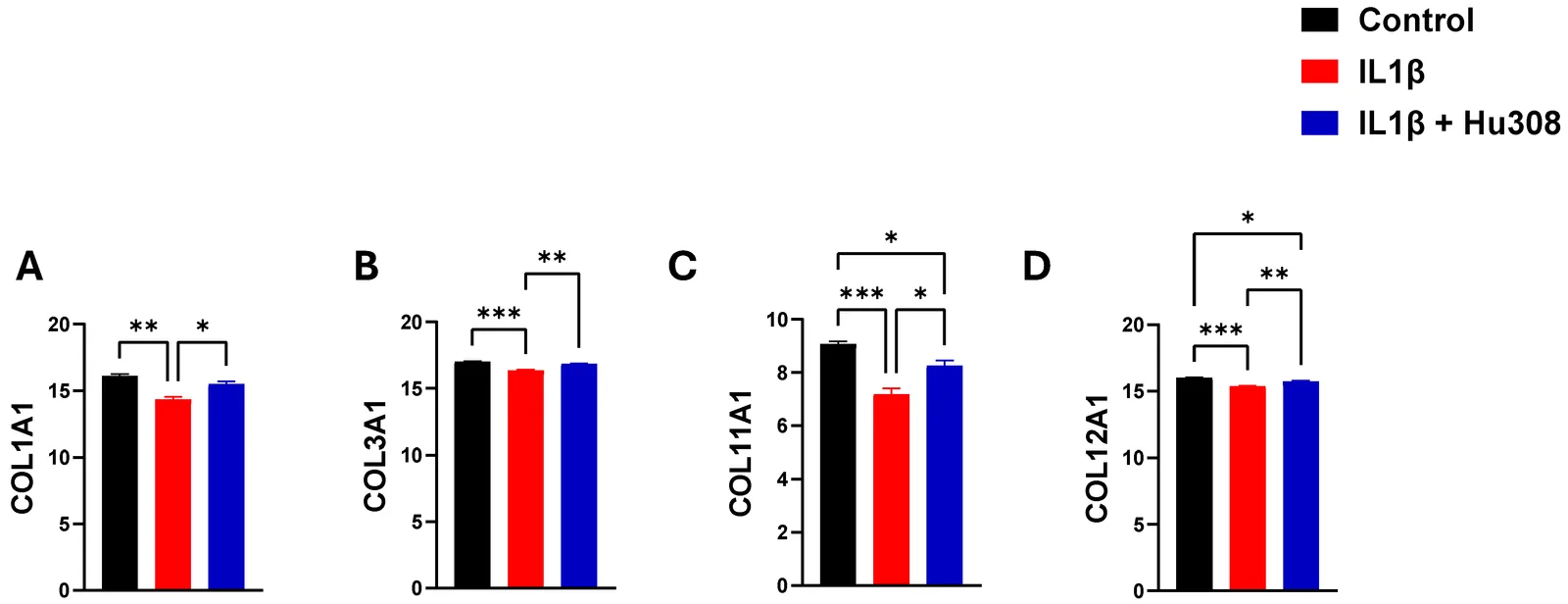
**HU-308 partially restores IL-1β-induced alterations in collagen gene expression in human gingival fibroblasts.** Human gingival fibroblasts (HGFs) were divided into three experimental groups: untreated Control (black bars), IL-1β (10 ng/mL)-stimulated cells (red bars), and cells treated with IL-1β followed by the selective CB2 receptor agonist HU-308 (10 μM; blue bars). Twenty-four hours after IL-1β stimulation, transcript levels of COL1A1, COL3A1, COL11A1, and COL12A1 were quantified. IL-1β stimulation significantly reduced the expression of collagen-associated genes, consistent with impaired extracellular matrix synthesis and tissue remodeling during periodontal inflammation (**A**–**D**). Treatment with HU-308 partially restored the expression of these collagen transcripts toward control levels, suggesting that CB2 receptor activation may preserve extracellular matrix homeostasis and limit IL-1β-induced connective tissue damage (**A**–**D**). Data are presented as mean ± SEM from three independent biological replicates (*n* = 3 per group). Statistical significance was determined by one-way ANOVA followed by Tukey’s multiple-comparison test. * *p* < 0.05, ** *p* < 0.01 and *** *p* < 0.001 was considered statistically significant.

To determine whether IL-1β alters extracellular matrix-associated gene expression, transcript levels of COL1A1, COL3A1, COL11A1, and COL12A1 were quantified in untreated HGFs, IL-1β-stimulated cells, and IL-1β-stimulated cells treated with HU-308 (Figure 4).

IL-1β stimulation significantly reduced the expression of all collagen genes examined compared with untreated controls. COL1A1 expression was significantly decreased following IL-1β stimulation compared with untreated controls (** *p* < 0.01; Figure 4A). Treatment with HU-308 significantly increased COL1A1 transcript levels relative to the IL-1β group (* *p* < 0.05; Figure 4A), restoring expression to levels that were not significantly different from untreated controls.

A similar pattern was observed for COL3A1. IL-1β significantly reduced COL3A1 expression compared with untreated controls (*** *p* < 0.001; Figure 4B), while HU-308 treatment significantly modulated COL3A1 transcript abundance relative to IL-1β-treated cells (** *p* < 0.01; Figure 4B). Following HU-308 treatment, COL3A1 expression was modulated to levels comparable with untreated controls.

In contrast, COL11A1 expression was markedly suppressed by IL-1β (*** *p* < 0.001 versus control; Figure 4C). Although HU-308 significantly increased COL11A1 expression relative to IL-1β-treated cells (* *p* < 0.05; Figure 4C), transcript levels remained significantly lower than those observed in untreated controls, indicating only partial recovery (* *p* < 0.05; Figure 4C).

Likewise, COL12A1 expression was significantly reduced following IL-1β stimulation (*** *p* < 0.001 versus control; Figure 4D). HU-308 significantly increased COL12A1 transcript abundance compared with IL-1β alone (** *p* < 0.01; Figure 4D); however, expression remained below control levels, demonstrating partial restoration rather than complete normalization (* *p* < 0.05; Figure 4D).

Collectively, these findings demonstrate that CB2 receptor activation reverses the suppressive effects of IL-1β on collagen gene expression. While HU-308 modulated COL1A1 and COL3A1 to levels comparable with untreated controls, recovery of COL11A1 and COL12A1 was incomplete, indicating differential regulation of collagen isoforms following CB2 activation.

### 2.5. HU-308 Modulates IL-1β-Associated Expression of NO/cGMP Signaling-Related Transcripts

**Figure 5:**
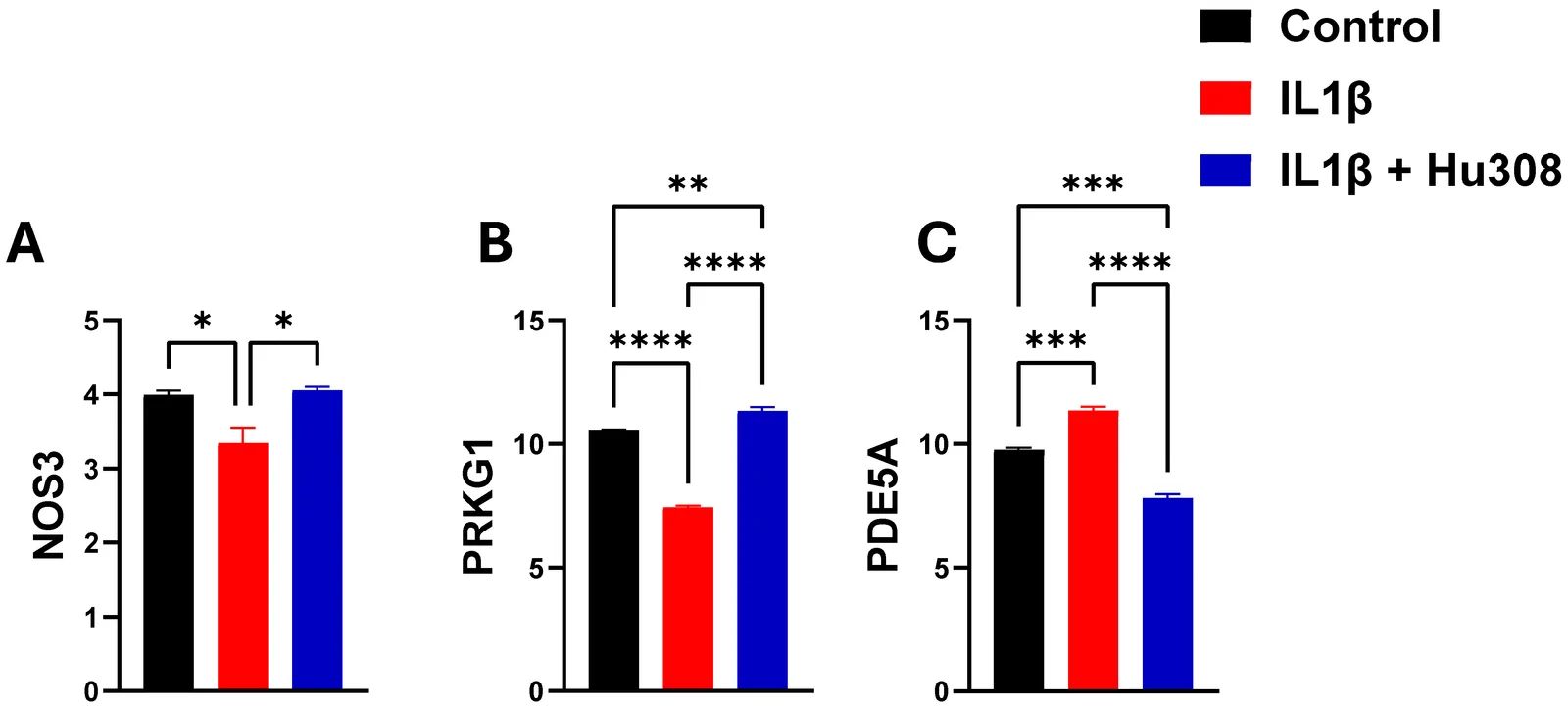
**HU-308 modulates IL-1β-induced nitric oxide and oxidative stress-related gene expression in human gingival fibroblasts.** Human gingival fibroblasts (HGFs) were divided into three experimental groups: untreated Control (black bars), IL-1β (10 ng/mL)-stimulated cells (red bars), and cells treated with IL-1β followed by the selective CB2 receptor agonist HU-308 (10 μM; blue bars). Twenty-four hours after IL-1β stimulation, transcript levels of NOS3, PRKG1 and PDE5A were quantified. IL-1β stimulation significantly altered the expression of genes involved in nitric oxide signaling, reflecting activation of redox-associated inflammatory pathways (**A**–**C**). Treatment with HU-308 modulated the IL-1β-induced expression of these genes, partially restoring their expression toward control levels and suggesting that CB2 receptor activation attenuates redox imbalance and nitric oxide signaling dysregulation associated with inflammatory activation (**A**–**C**). Data are presented as mean ± SEM from three independent biological replicates (*n* = 3 per group). Statistical significance was determined by one-way ANOVA followed by Tukey’s multiple-comparison test. * *p* < 0.05, ** *p* < 0.01, *** *p* < 0.001, and **** *p* < 0.0001 was considered statistically significant.

To investigate whether CB2 receptor activation influences genes involved in nitric oxide (NO)/cGMP signaling, transcript levels of NOS3, PRKG1, and PDE5A were quantified in untreated HGFs, IL-1β-stimulated cells, and IL-1β-stimulated cells treated with HU-308 (Figure 5).

The endothelial nitric oxide synthase gene NOS3 was significantly downregulated following IL-1β stimulation compared with untreated controls (* *p* < 0.05; Figure 5A). Treatment with HU-308 significantly increased NOS3 expression relative to the IL-1β group (* *p* < 0.05; Figure 5A), restoring transcript levels to values that were not significantly different from untreated controls.

Protein Kinase type I expression was significantly reduced by IL-1β compared with control cells (**** *p* < 0.0001; Figure 5B). HU-308 treatment significantly increased PRKG1 transcript abundance relative to the IL-1β group (**** *p* < 0.0001; Figure 5B). Moreover, PRKG1 expression in the HU-308-treated group was significantly higher than that observed in untreated controls (** *p* < 0.01; Figure 5B), indicating that HU-308 treatment not only reversed the IL-1β-induced suppression but also elevated PRKG1 expression above basal levels.

Conversely, PDE5A expression was significantly increased following IL-1β stimulation compared with untreated controls (*** *p* < 0.001; Figure 5C). Treatment with HU-308 significantly reduced PDE5A expression relative to the IL-1β group (**** *p* < 0.0001; Figure 5C). Notably, PDE5A transcript levels following HU-308 treatment were significantly lower than those observed in untreated controls (*** *p* < 0.001; Figure 5C), indicating that CB2 receptor activation suppressed PDE5A expression below basal levels.

Collectively, these findings demonstrate that HU-308 treatment was associated with differential regulation of NOS3, PRKG1, and PDE5A transcript expression under IL-1β-stimulated conditions. Because NO production, cGMP abundance, and enzymatic activity were not measured, these transcriptional changes should not be interpreted as direct evidence of restored NO/cGMP signaling.

### 2.6. HU-308 Modulates IL-1β-Associated Expression of Selected Solute Carrier Transporter Transcripts

**Figure 6:**
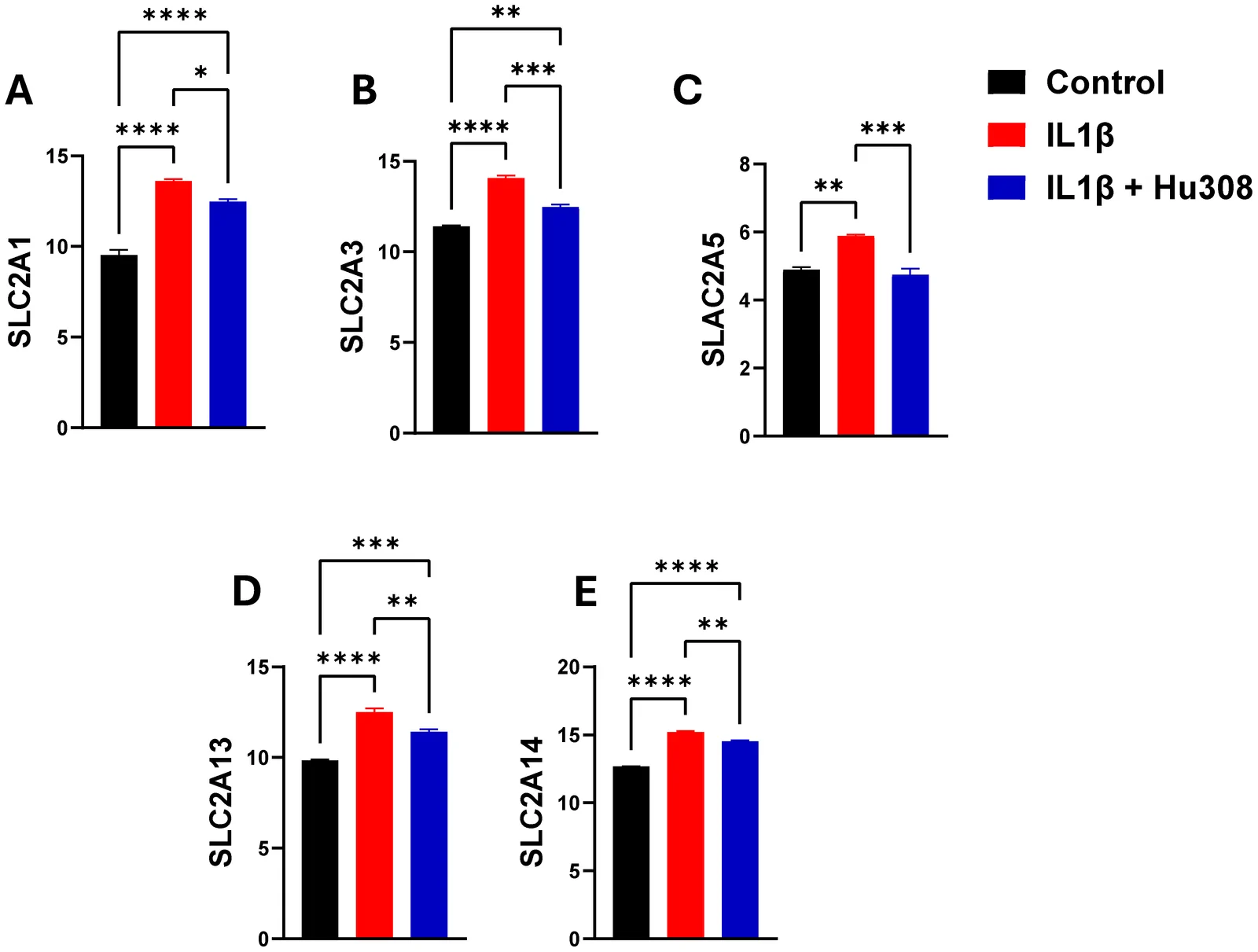
**HU-308 modulates IL-1β-associated changes in solute carrier transporter transcript expression in human gingival fibroblasts.** Human gingival fibroblasts (HGFs) were divided into three experimental groups: untreated Control (black bars), IL-1β (10 ng/mL)-stimulated cells (red bars), and cells treated with IL-1β followed by the selective CB2 receptor agonist HU-308 (10 μM; blue bars). Twenty-four hours after IL-1β stimulation, transcript levels of SLC2A1, SLC2A3, SLC2A5, SLC5A13, and SLC2A14 were quantified. IL-1β stimulation significantly altered the expression of SLC2A1, SLC2A3, SLC2A5, SLC2A13, and SLC2A14, and HU-308 treatment significantly modified several of these IL-1β-associated transcriptional responses (**A**–**E**). These findings demonstrate changes in transporter-associated transcript abundance but do not establish corresponding alterations in substrate uptake, glucose utilization, glycolytic activity, or cellular metabolic flux. Data are presented as mean ± SEM from three independent biological replicates (*n* = 3 per group). Statistical significance was determined by one-way ANOVA followed by Tukey’s multiple-comparison test. * *p* < 0.05, ** *p* < 0.01, *** *p* < 0.001, and **** *p* < 0.0001 was considered statistically significant.

To investigate whether inflammatory stimulation alters the expression of selected solute carrier transporters, transcript levels of SLC2A1, SLC2A3, SLC2A5, SLC2A13, and SLC2A14 were examined in untreated HGFs, IL-1β-stimulated cells, and IL-1β-stimulated cells treated with HU-308 (Figure 6). These transporters differ in their established substrate specificity and biological functions; therefore, changes in their transcript abundance were interpreted as transporter-associated transcriptional responses rather than direct evidence of altered glucose metabolism or metabolic reprogramming.

IL-1β stimulation significantly altered the expression of several of these transporter-associated transcripts, and HU-308 treatment modified a subset of these IL-1β-associated responses. Because glucose uptake, substrate transport, glycolytic activity, and metabolic flux were not directly measured, the functional metabolic consequences of these transcriptional changes remain to be determined. SLC2A1 expression was significantly increased following IL-1β stimulation compared with untreated controls (**** *p* < 0.0001; Figure 6A). Treatment with HU-308 significantly reduced SLC2A1 transcript levels relative to the IL-1β group (* *p* < 0.05; Figure 6A). However, expression remained significantly higher than that observed in untreated controls, indicating partial attenuation of the inflammatory response (**** *p* < 0.0001; Figure 6A).

Similarly, SLC2A3 was significantly upregulated following IL-1β stimulation compared with untreated cells (**** *p* < 0.0001; Figure 6B). HU-308 significantly suppressed SLC2A3 expression relative to IL-1β-treated cells (*** *p* < 0.001; Figure 6B), although transcript levels remained significantly elevated compared with untreated controls (** *p* < 0.01; Figure 6B).

IL-1β significantly increased SLC2A5 expression compared with untreated controls (** *p* < 0.01; Figure 6C). Treatment with HU-308 significantly reduced SLC2A5 transcript levels relative to the IL-1β group (*** *p* < 0.001; Figure 6C), restoring expression to levels that were no longer significantly different from untreated controls.

Likewise, SLC5A13 expression was significantly increased following IL-1β stimulation compared with untreated controls (**** *p* < 0.0001; Figure 6D). HU-308 significantly reduced SLC5A13 transcript levels relative to the IL-1β group (** *p* < 0.01; Figure 6D), partially reversing the inflammatory induction while remaining significantly higher than basal expression (*** *p* < 0.001; Figure 6D).

Finally, SLC2A14 expression was significantly elevated after IL-1β stimulation compared with untreated controls (**** *p* < 0.01; Figure 6E). HU-308 treatment significantly decreased SLC2A14 expression relative to IL-1β-treated cells (** *p* < 0.01; Figure 6E), indicating attenuation of the IL-1β-induced response, although expression remained above that observed in untreated cells (**** *p* < 0.0001; Figure 6E).

Collectively, these findings demonstrate that IL-1β stimulation is associated with altered expression of several solute carrier transporter transcripts in HGFs and that HU-308 treatment significantly modifies a subset of these responses. Because the transporters examined have different substrate specificities and because substrate uptake and cellular metabolic flux were not measured, these results should be interpreted as transcriptional alterations in transporter-associated genes rather than evidence of functional metabolic reprogramming.

### 2.7. G Protein-Coupled Receptor Transcript Expression Is Differentially Modulated by IL-1β and HU-308

**Figure 7:**
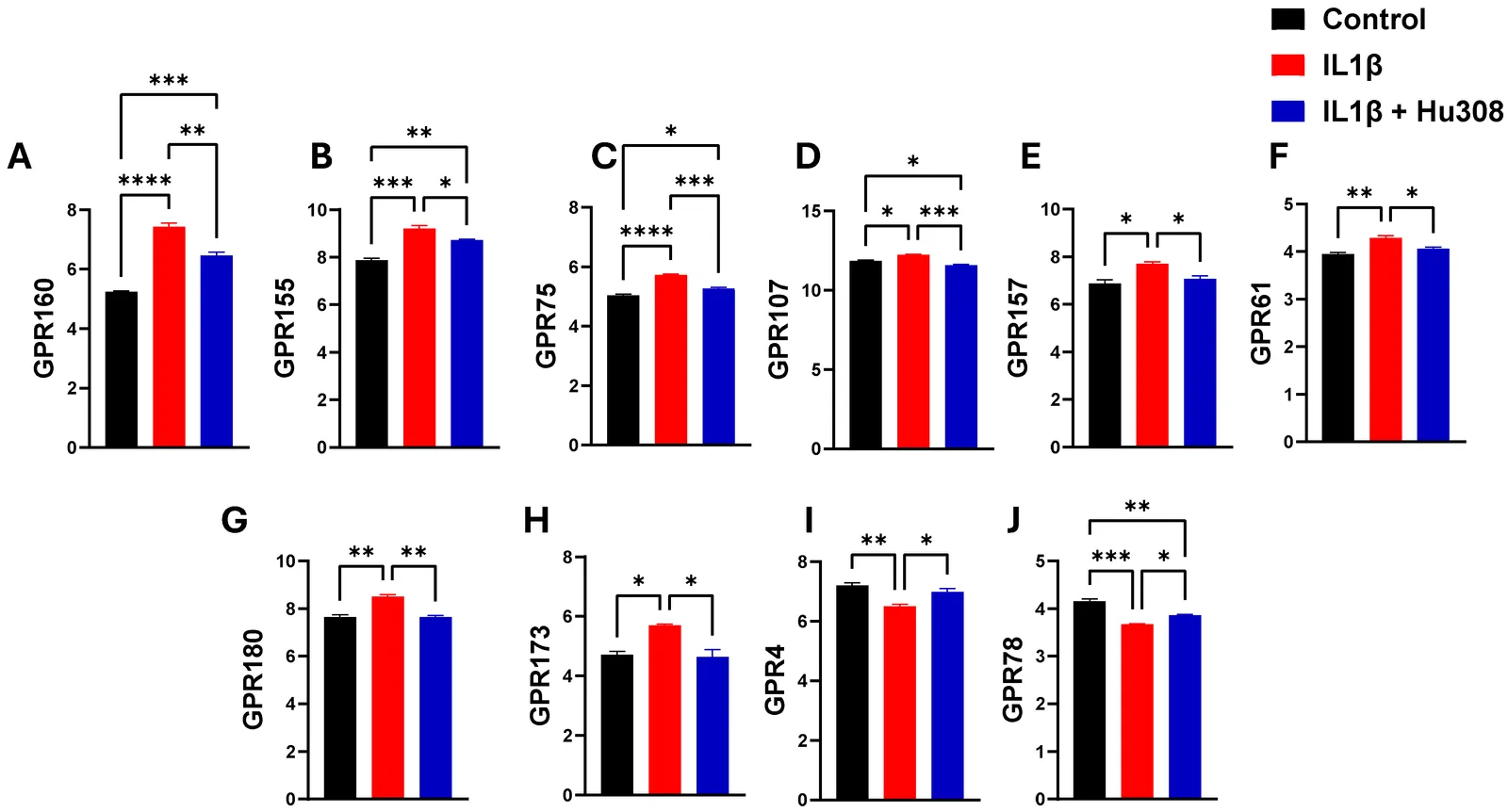
**GPCR-related transcript modulation by IL-1β and HU-308 treatment in human gingival fibroblasts.** Human gingival fibroblasts (HGFs) were divided into three experimental groups: untreated Control (black bars), IL-1β (10 ng/mL)-stimulated cells (red bars), and cells treated with IL-1β followed by the selective CB2 receptor agonist HU-308 (10 μM; blue bars). Twenty-four hours after IL-1β stimulation, transcript levels of GPR160, GPR180, GPR157, GPR61, GPR173, GPR155, GPR107, GPR75, GPR78, and GPR4 were quantified. IL-1β stimulation significantly altered the expression of multiple GPCR-related genes, including increased expression of GPR160, GPR180, GPR157, GPR61, GPR173, GPR155, GPR107, and GPR75, while reducing the expression of GPR78 and GPR4, indicating inflammatory remodeling of GPCR-associated signaling pathways in human gingival fibroblasts (**A**–**J**). Treatment with HU-308 modified several IL-1β-associated changes in GPCR-related transcript expression (**A**–**J**). These changes represent transcriptional associations and should not be interpreted as evidence of altered receptor activity or direct functional cross-talk with CB2. Data are presented as mean ± SEM from three independent biological replicates (*n* = 3 per group). Statistical significance was determined by one-way ANOVA followed by Tukey’s multiple-comparison test. * *p* < 0.05, ** *p* < 0.01, *** *p* < 0.001, and **** *p* < 0.0001 was considered statistically significant.

To investigate whether inflammatory stimulation and CB2 receptor activation influence G protein-coupled receptor (GPCR)-related transcriptional responses, the expression of GPR160, GPR180, GPR157, GPR61, GPR173, GPR155, GPR107, GPR78, GPR4, and GPR75 was quantified in untreated human gingival fibroblasts (HGFs), IL-1β-stimulated cells, and IL-1β-stimulated cells subsequently treated with the selective CB2 receptor agonist HU-308 (Figure 7).

IL-1β stimulation significantly increased the expression of GPR160 (**** *p* < 0.0001; Figure 7A). Treatment with HU-308 significantly reduced GPR160 expression compared with the IL-1β group (** *p* < 0.01; Figure 7A), although transcript levels remained significantly higher than those observed in untreated controls (*** *p* < 0.001; Figure 7A), indicating partial reversal of the inflammatory response.

Expression of GPR155, GPR75 and GPR107 was also significantly elevated following IL-1β stimulation (*** *p* < 0.001, **** *p* < 0.0001, * *p* < 0.05, respectively; Figure 7B–D). Although HU-308 significantly reduced the expression of the 3 genes compared with IL-1β treatment alone (* *p* < 0.05, and *** *p* < 0.001, respectively Figure 7B–D), transcript levels remained significantly higher than those of untreated controls, indicating only partial attenuation of the inflammatory response (** *p* < 0.01, and * *p* < 0.05, respectively; Figure 7B–D).

A similar pattern was observed for GPR157, GPR61, GPR180, and GPR173 (Figure 7E–H). IL-1β significantly increased the expression of each transcript relative to untreated controls (* *p* < 0.05, ** *p* < 0.01, respectively; Figure 7E–H), whereas HU-308 significantly reduced their expression compared with IL-1β-treated cells. Following HU-308 treatment, transcript levels approached control values and were not statistically different from the untreated control under the tested condition (Figure 7E–H).

In contrast, IL-1β significantly suppressed the expression of GPR78 and GPR4 (*** *p* < 0.001, respectively; Figure 7I,J). HU-308 treatment modulated the expression of both receptors relative to IL-1β-treated cells (* *p* < 0.05, respectively; Figure 7I,J). For GPR4, expression returned to a level that was no longer significantly different from untreated controls (Figure 7I), whereas GPR78 showed only partial recovery and remained significantly below control level (** *p* < 0.001; Figure 7J).

Overall, IL-1β induced broad remodeling of GPCR-related gene expression in HGFs, characterized by increased expression of GPR160, GPR180, GPR157, GPR61, GPR173, GPR155, GPR107, and GPR75, together with decreased expression of GPR78 and GPR4. Activation of CB2 receptors by HU-308 partially or completely reversed many of these transcriptional alterations, supporting a broader role for CB2 signaling in modulating GPCR-associated transcriptional networks during inflammatory activation of gingival fibroblasts.

## 3. Discussion

The present findings should be interpreted within the context of the experimental design, which examined a single concentration of IL-1β, a single concentration of HU-308, and one 24-h endpoint. Therefore, the observed transcriptional changes represent responses under this specific experimental condition and do not establish dose-dependent effects, temporal kinetics, or the durability of the HU-308-associated response.

Periodontitis is a chronic inflammatory disease characterized by a dysregulated host immune response that progressively destroys the gingival connective tissue, periodontal ligament, and alveolar bone supporting the dentition [21,22,23]. Although bacterial dysbiosis initiates disease development, tissue destruction is primarily driven by an exaggerated host inflammatory response involving resident stromal cells, infiltrating immune cells, and a complex network of inflammatory mediators [2,8]. Among resident periodontal cells, gingival fibroblasts play a pivotal role in orchestrating these responses [11,24]. In addition to maintaining extracellular matrix homeostasis, gingival fibroblasts actively respond to inflammatory stimuli by producing cytokines, chemokines, matrix metalloproteinases, and other mediators that regulate immune-cell recruitment, connective tissue remodeling, angiogenesis, and tissue repair [8,11,25]. Consequently, modulation of fibroblast inflammatory responses has emerged as an attractive therapeutic strategy for limiting periodontal tissue destruction while preserving normal tissue homeostasis [10,26].

Cannabinoid receptor type 2 (CB2) has attracted increasing attention as a potential therapeutic target because of its predominantly immunomodulatory actions and minimal psychoactive effects compared with CB1 receptor activation [27,28,29,30]. Previous studies have demonstrated that CB2 activation attenuates inflammatory signaling in multiple cell types by suppressing NF-κB and MAPK-dependent pathways, resulting in reduced cytokine production, diminished oxidative stress, and improved tissue repair [15,31,32]. However, the extent to which CB2 activation coordinately regulates multiple inflammatory pathways in human gingival fibroblasts has remained poorly understood. Most previous investigations have focused on individual cytokines or isolated signaling pathways, providing only a limited understanding of how CB2 activation influences the complex molecular networks that collectively drive periodontal inflammation.

The present study provides a comprehensive transcriptomic evaluation of IL-1β-induced inflammatory responses in human gingival fibroblasts and demonstrates that pharmacological activation of CB2 with HU-308 modulates multiple interconnected biological pathways rather than a single inflammatory axis. IL-1β stimulation produced coordinated transcriptional changes characterized by increased expression of pro-inflammatory cytokines, chemokines, and matrix metalloproteinases, together with suppression of collagen-associated and anti-inflammatory genes. These inflammatory alterations were accompanied by dysregulation of nitric oxide signaling and glucose transporter-associated metabolic pathways, illustrating the broad biological consequences of inflammatory activation in gingival fibroblasts. Importantly, HU-308 consistently attenuated these transcriptional changes across all functional categories examined, indicating that CB2 activation exerts pleiotropic effects on inflammatory, structural, redox, and metabolic pathways.

Rather than acting as a general suppressor of cellular activity, HU-308 selectively regulated IL-1β-responsive genes. Several inflammatory mediators, including IL1B, IL11, IL23A, IL24, IL10, COL1A1, COL3A1, and NOS3, were modulated to levels comparable with untreated controls, whereas other genes, including IL6, IL33, MMP1, MMP3, MMP10, COL11A1, COL12A1, SLC2A1, SLC2A3, SLC2A13, and SLC2A14, demonstrated partial normalization. In contrast, PRKG1 and PDE5A exhibited responses that extended beyond basal expression levels, suggesting differential sensitivity of downstream signaling pathways to CB2 activation. These observations indicate that CB2 signaling does not function as a simple on/off switch for inflammation but instead differentially regulates multiple transcriptional networks according to their biological context.

Accordingly, the following sections discuss how coordinated regulation of inflammatory cytokines, chemokines, extracellular matrix remodeling, nitric oxide signaling, and metabolic pathways may collectively contribute to the anti-inflammatory and tissue-preserving effects of CB2 activation in gingival fibroblasts.

### 3.1. CB2 Receptor Activation Attenuates the Cytokine–Chemokine Network Driving Periodontal Inflammation

The first major finding of the present study is that CB2 receptor activation broadly suppresses the inflammatory cytokine–chemokine axis induced by IL-1β in human gingival fibroblasts. Rather than regulating isolated inflammatory mediators, HU-308 simultaneously modulated multiple cytokines and chemokines that collectively orchestrate immune-cell activation, inflammatory amplification, and leukocyte recruitment. This coordinated regulation is particularly relevant because periodontal disease is no longer viewed as the consequence of individual cytokines but rather as the result of a highly interconnected inflammatory network in which resident gingival fibroblasts actively participate in initiating and sustaining chronic inflammation.

Among the cytokines examined, IL-6 and IL1β represent two of the principal mediators driving periodontal disease progression [8,33]. IL-1β functions as a master pro-inflammatory cytokine that stimulates fibroblasts, epithelial cells, and infiltrating leukocytes to produce additional inflammatory mediators, thereby establishing a positive-feedback loop that perpetuates tissue inflammation [34,35]. Likewise, IL-6 promotes leukocyte activation, B-cell differentiation, osteoclastogenesis, and alveolar bone resorption and has consistently been associated with disease severity and clinical attachment loss in patients with periodontitis [36,37,38,39]. The marked suppression of both IL1β and IL-6 following HU-308 treatment therefore suggests interruption of one of the major inflammatory amplification pathways that maintain chronic periodontal inflammation.

Additional cytokines identified in the present study further emphasize the broad anti-inflammatory effects of CB2 activation. IL23A is a critical regulator of the IL-23/Th17 axis, which promotes sustained production of IL-17 and enhances neutrophil recruitment, osteoclast activation, and chronic tissue destruction [40,41,42,43]. IL33, an epithelial- and stromal-derived alarmin, is released following tissue injury and amplifies both innate and adaptive immune responses by activating mast cells, macrophages, and T lymphocytes [44,45,46]. IL11, although traditionally associated with tissue remodeling and fibrosis, is increasingly recognized as a regulator of inflammatory fibroblast activation and chronic stromal remodeling [47]. IL24 has likewise been implicated in chronic inflammatory diseases through the regulation of cytokine production and immune cell activation [48,49]. The ability of HU-308 to normalize the expression of several of these cytokines indicates that CB2 activation influences multiple inflammatory pathways simultaneously rather than targeting a single upstream mediator.

Equally important was the restoration of the anti-inflammatory cytokine IL-10. IL-10 is widely recognized as one of the most potent endogenous suppressors of inflammatory cytokine production, functioning to inhibit NF-κB activation, reduce macrophage activation, and limit excessive immune responses [50,51,52,53]. Reduced IL-10 expression has been associated with increased susceptibility to periodontal tissue destruction, whereas restoration of IL-10 promotes resolution of inflammation and preservation of periodontal tissues [8,54]. Restoration of IL-10 following HU-308 treatment therefore indicates that CB2 activation not only suppresses inflammatory mediators but also promotes endogenous pathways involved in controlling inflammation and maintaining tissue homeostasis.

The reduction in inflammatory cytokine expression was accompanied by coordinated suppression of multiple chemokines, providing further evidence that CB2 activation interrupts the inflammatory cascade at several levels. Chemokines function as the principal mediators of leukocyte trafficking and are responsible for recruiting inflammatory cells from the circulation into periodontal tissues, where they sustain cytokine production and tissue injury [55,56]. CXCL5 (RANTES) is a potent chemoattractant for T lymphocytes, monocytes, dendritic cells, and osteoclast precursors and has been associated with alveolar bone loss and periodontal disease progression [57,58,59]. CXCL8 recruits monocytes, macrophages, activated T cells, and natural killer cells, thereby amplifying chronic inflammatory responses [60,61]. Although less extensively characterized in periodontitis, CXCL16 contributes to leukocyte migration and inflammatory cell activation, suggesting a role in maintaining persistent inflammatory infiltrates [62,63]. CCL20 plays a pivotal role in recruiting CCR6-positive Th17 cells and immature dendritic cells, thereby linking innate and adaptive immunity during periodontal inflammation [64,65]. In contrast, CCL28 contributes to mucosal immune homeostasis by recruiting IgA-secreting plasma cells and memory T lymphocytes but is also upregulated during chronic inflammatory conditions [66,67,68,69]. The coordinated suppression of these chemokines following HU-308 treatment suggests that CB2 activation may reduce recruitment of inflammatory cells into periodontal tissues, thereby interrupting one of the major mechanisms sustaining chronic inflammation.

Importantly, the coordinated modulation of cytokines and chemokines observed in the present study suggests a sequential biological response rather than independent transcriptional events. Suppression of IL-1B, IL-6, and IL-23 signaling would be expected to reduce downstream chemokine production, thereby limiting leukocyte recruitment and inflammatory amplification within periodontal tissues. Consequently, attenuation of this cytokine–chemokine network provides a mechanistic framework for understanding the subsequent reductions in matrix metalloproteinase expression and restoration of extracellular matrix-associated genes observed in the present study, linking early inflammatory signaling directly to structural preservation of periodontal connective tissues.

### 3.2. CB2 Receptor Activation Preserves Extracellular Matrix Homeostasis by Coordinately Regulating Matrix Metalloproteinases and Collagen Synthesis

Because inflammatory cytokines and chemokines are potent inducers of extracellular matrix degradation [70,71], attenuation of the inflammatory response would be expected to influence connective tissue remodeling. Consistent with this concept, suppression of the cytokine–chemokine network by HU-308 was accompanied by coordinated downregulation of matrix metalloproteinases and restoration of collagen-associated gene expression. These findings suggest that CB2 activation not only limits inflammatory signaling but also preserves the structural integrity of periodontal connective tissues by simultaneously reducing matrix degradation and promoting extracellular matrix maintenance.

Matrix metalloproteinases (MMPs) are among the principal mediators of connective tissue destruction during periodontitis [72,73]. Although tightly regulated under physiological conditions, excessive MMP production contributes to degradation of the periodontal ligament and gingival connective tissue, ultimately leading to attachment loss and disease progression [72,74,75]. In the present study, IL-1β markedly increased the expression of MMP1, MMP3, and MMP10, all of which were significantly attenuated following HU-308 treatment.

Each of these metalloproteinases contributes to tissue destruction through distinct but complementary mechanisms. MMP1 initiates degradation of fibrillar collagens, particularly type I and type III collagen, which represent the major structural components of gingival connective tissue and the periodontal ligament [76,77,78]. MMP3 possesses broad proteolytic activity against extracellular matrix proteins and, importantly, activates several additional matrix metalloproteinases, thereby amplifying proteolytic cascades within inflamed periodontal tissues [79,80]. MMP10 exhibits similar substrate specificity and contributes to extracellular matrix remodeling, inflammatory tissue destruction, and wound remodeling. Increased expression of these enzymes has consistently been associated with clinical attachment loss, connective tissue degradation, and progression of periodontal disease. Their coordinated suppression following HU-308 treatment therefore suggests that CB2 activation interrupts one of the major effector mechanisms responsible for connective tissue destruction.

Suppression of matrix metalloproteinases alone, however, is insufficient to preserve tissue integrity if extracellular matrix synthesis remains impaired. Accordingly, the accompanying restoration of collagen gene expression represents an equally important finding of the present study. Collagens constitute the primary structural scaffold of periodontal connective tissues and are essential for maintaining tissue architecture, tensile strength, and mechanical stability [81]. COL1A1 encodes type I collagen, the predominant collagen within gingival connective tissue and the periodontal ligament [82], whereas COL3A1 encodes type III collagen, which is particularly important during wound healing, early matrix deposition, and tissue repair [83]. The complete restoration of both COL1A1 and COL3A1 expression following HU-308 treatment therefore suggests recovery of the principal collagen framework required for maintenance and regeneration of periodontal connective tissue.

The remaining collagen isoforms exhibited a more modest response to CB2 activation. COL11A1 regulates collagen fibril assembly and organization [84], thereby influencing fibril diameter and extracellular matrix architecture, whereas COL12A1 belongs to the fibril-associated collagens with interrupted triple helices (FACIT) family and functions to stabilize collagen fibrils and facilitate interactions between collagen fibers and surrounding matrix components [85,86]. Although HU-308 significantly increased the expression of both genes compared with IL-1β-treated cells, neither transcript returned to a level that was not statistically different from the untreated control at the 24-h endpoint. This differential response suggests that individual collagen isoforms exhibit varying sensitivity to inflammatory signaling and CB2-mediated regulation, reflecting the complexity of extracellular matrix remodeling during inflammation.

Importantly, the reciprocal regulation of matrix metalloproteinases and collagen genes observed in the present study indicates coordinated preservation of extracellular matrix homeostasis rather than isolated modulation of individual structural proteins. This should be interpreted as transcript-level evidence of a shift away from a matrix-degradative fibroblast phenotype. Additional protein-level or functional validation, such as MMP activity assays and collagen deposition studies, would be needed before concluding that HU-308 directly preserves extracellular matrix integrity. Suppression of inflammatory cytokines and chemokines would be expected to reduce MMP induction, thereby limiting collagen degradation, while simultaneous restoration of collagen synthesis promotes reconstruction of the extracellular matrix. Together, these complementary effects suggest that CB2 activation shifts gingival fibroblasts from a tissue-destructive phenotype toward one that favors structural preservation and repair. This coordinated regulation of extracellular matrix turnover may represent one of the principal mechanisms through which CB2 activation limits connective tissue destruction during periodontal inflammation.

Extracellular matrix remodeling is closely linked to oxidative stress and nitric oxide signaling [87,88]. These findings also provide a mechanistic basis for the alterations observed in the NO/cGMP pathway. Persistent inflammatory signaling and matrix degradation increase oxidative stress within periodontal tissues, whereas disruption of nitric oxide homeostasis further amplifies inflammation and impairs tissue repair. The coordinated modulation of these pathways by HU-308 therefore suggests that CB2 modulation extends beyond extracellular matrix preservation to broader regulation of cellular signaling networks involved in periodontal homeostasis.

### 3.3. CB2 Receptor Activation Modulates Nitric Oxide/cGMP Signaling and Redox Homeostasis During Periodontal Inflammation

Beyond its effects on inflammatory mediator production and extracellular matrix remodeling, the present study demonstrates that CB2 modulation also influences genes involved in nitric oxide (NO)/cyclic guanosine monophosphate (cGMP) signaling, suggesting that HU-308 regulates pathways critical for vascular homeostasis, oxidative stress, and inflammatory signaling. Increasing evidence indicates that disturbances in the NO/cGMP axis contribute to the pathogenesis of periodontitis by promoting endothelial dysfunction, excessive reactive oxygen species (ROS) production, impaired tissue perfusion, and persistent inflammatory activation [89,90,91,92,93]. Thus, restoration of this pathway may represent an additional mechanism through which CB2 modulation limits periodontal tissue injury.

Nitric oxide is a key signaling molecule that regulates vascular tone, leukocyte adhesion, angiogenesis, and inflammatory responses [94,95]. Within periodontal tissues, physiological NO production contributes to maintenance of the microvasculature, regulation of local blood flow, and resolution of inflammation [96]. NOS3 (endothelial nitric oxide synthase) is the principal enzyme responsible for constitutive NO production in vascular endothelial cells and has also been detected in periodontal tissues, where it contributes to tissue homeostasis and wound healing [97,98,99]. Reduced NOS3 expression has been associated with impaired endothelial function, diminished NO bioavailability, and increased oxidative stress [100]. In the present study, IL-1β significantly suppressed NOS3 expression, whereas HU-308 modulated transcript levels to those observed in untreated cells. Restoration of NOS3 expression suggests that HU-308 treatment may improve NO bioavailability and help preserve vascular and inflammatory homeostasis during periodontal inflammation.

Nitric oxide exerts many of its biological effects through activation of soluble guanylate cyclase and subsequent production of cGMP [101,102], which activates protein kinase G (PRKG1) [103,104]. PRKG1 regulates numerous downstream processes, including vascular relaxation, inhibition of inflammatory signaling, maintenance of endothelial barrier integrity, and protection against oxidative stress. Interestingly, HU-308 not only reversed the IL-1β-induced reduction in PRKG1 expression but also increased transcript levels beyond those observed in untreated controls. This finding may indicate enhanced activation of the NO/cGMP signaling cascade following CB2 stimulation and suggests that restoration of NO signaling extends beyond recovery of NOS3 expression alone.

In contrast, PDE5A functions as the principal phosphodiesterase responsible for degradation of cGMP [105,106], thereby limiting the duration and magnitude of NO-dependent signaling. Increased PDE5 activity has been associated with endothelial dysfunction, impaired vasodilation, and chronic inflammatory diseases through excessive degradation of cGMP [107,108]. Consistent with these observations, IL-1β significantly increased PDE5A expression, whereas HU-308 markedly suppressed PDE5A expression to levels below those observed in untreated controls. The simultaneous restoration of NOS3, enhancement of PRKG1, and suppression of PDE5A collectively suggest that HU-308 treatment favors preservation of NO/cGMP signaling by simultaneously increasing NO production, enhancing downstream signal transduction, and limiting cGMP degradation.

The observed modulation of the NO/cGMP pathway is also closely linked to oxidative stress, an important driver of periodontal disease progression. Chronic inflammatory stimulation promotes excessive ROS production, which further activates NF-κB signaling, enhances cytokine production, increases matrix metalloproteinase activity, and suppresses extracellular matrix synthesis, thereby establishing a self-perpetuating cycle of tissue destruction [109]. Previous studies have demonstrated that CB2 activation reduces oxidative stress by inhibiting ROS-generating pathways and enhancing endogenous antioxidant responses [110]. Although oxidative stress was not directly measured in the present study, the coordinated regulation of NOS3, PRKG1, and PDE5A is consistent with improved redox homeostasis and reduced oxidative signaling following HU-308 treatment.

Oxidative stress may provide an additional mechanistic context for the observed transcriptional changes in NO/cGMP-associated genes. During inflammatory activation, increased production of reactive oxygen species (ROS), including ROS generated by NADPH oxidases, can reduce nitric oxide bioavailability and contribute to disruption of redox-sensitive signaling pathways. Conversely, the nuclear factor erythroid 2-related factor 2 (Nrf2) pathway serves as an important cellular defense mechanism by promoting the expression of antioxidant and cytoprotective genes that limit oxidative and inflammatory stress. Previous studies have demonstrated interactions among cannabinoid signaling, oxidative stress, NADPH oxidase activity, and Nrf2-associated antioxidant responses, suggesting that these pathways may provide additional mechanisms through which cannabinoid-related signaling influences inflammatory cellular responses. However, the present study did not directly assess ROS production, NADPH oxidase activity, Nrf2 activation, or antioxidant capacity. Therefore, whether these redox-regulatory mechanisms contribute to the HU-308-associated transcriptional changes observed in HGFs remains to be determined and represents an important direction for future investigation.

Collectively, these findings suggest that CB2 modulation extends beyond suppression of inflammatory cytokines and preservation of extracellular matrix homeostasis to include restoration of signaling pathways that regulate vascular function and oxidative balance. Because nitric oxide signaling influences inflammatory-cell recruitment, fibroblast activation, collagen synthesis, and tissue repair, modulation of the NO/cGMP axis may represent an important mechanism linking the anti-inflammatory and tissue-protective effects of HU-308 observed throughout the present study. Furthermore, restoration of cellular signaling pathways that regulate energy demand and stress adaptation provides a mechanistic bridge to the metabolic alterations in glucose transporter expression discussed in the following section.

### 3.4. HU-308 Modulates Transporter Expression Associated with Inflammatory Metabolic Adaptation

Inflammation is increasingly recognized as being closely linked to cellular metabolism, as inflammatory activation can alter nutrient utilization and cellular energetic demands required to support cytokine production, extracellular matrix remodeling, proliferation, and tissue repair. Gingival fibroblasts are metabolically responsive stromal cells that adapt to changes in their inflammatory microenvironment, and alterations in nutrient transporter expression may represent one component of this adaptive response. In the present study, IL-1β altered the expression of several members of the SLC2A transporter family, suggesting that inflammatory stimulation is accompanied by transcriptional changes in genes involved in cellular substrate transport. However, because transporter activity and metabolic flux were not directly measured, these changes should be interpreted as transcriptional adaptations associated with inflammation rather than direct evidence of metabolic reprogramming.

Among the transporters examined, SLC2A1 (GLUT1) and SLC2A3 (GLUT3) are particularly relevant to the relationship between inflammation and cellular glucose metabolism. GLUT1 is a major facilitative glucose transporter responsible for basal glucose uptake and can be induced during inflammatory activation to support increased cellular glucose requirements [111,112]. Increased GLUT1 expression has also been associated with NF-κB signaling, cytokine production, oxidative stress, and persistent inflammatory responses in immune and stromal cells [111,113,114]. Similarly, GLUT3 is a high-affinity glucose transporter capable of maintaining glucose uptake under conditions of increased cellular demand or reduced glucose availability [115]. Although initially characterized predominantly in neuronal tissues, GLUT3 has increasingly been implicated in inflammatory cell activation and metabolic adaptation [116,117]. Thus, the observed modulation of SLC2A1 and SLC2A3 by HU-308 may reflect an influence on transporter-associated responses accompanying inflammatory activation. Whether these transcriptional changes result in altered glucose uptake or glycolytic activity, however, cannot be determined from the present data.

SLC2A5 (GLUT5) provides an additional dimension to these findings because its primary substrate is fructose rather than glucose. GLUT5 has increasingly been investigated in the context of inflammatory metabolism and altered fructose utilization has been associated with oxidative stress, inflammatory signaling, and extracellular matrix remodeling in several chronic inflammatory conditions [118,119,120,121]. The modulation of SLC2A5 expression by HU-308 therefore suggests that the transcriptional response may extend beyond conventional glucose transport. Nevertheless, normalization of SLC2A5 transcript abundance should not be interpreted as evidence that fructose metabolism itself was restored, as substrate uptake and metabolic utilization were not directly assessed.

The regulation of SLC2A13 (GLUT13/HMIT) is also noteworthy because this transporter differs functionally from conventional GLUT family members. SLC2A13 primarily functions as a proton-coupled myo-inositol transporter rather than a glucose transporter [122]. Myo-inositol is an important precursor for phosphoinositides and inositol phosphates involved in intracellular calcium signaling, membrane trafficking, and multiple cellular signaling pathways. Although the role of SLC2A13 in periodontal tissues remains largely unexplored, changes in its expression raise the possibility that inflammatory stimulation and HU-308 treatment may influence substrate-transport processes extending beyond glucose metabolism. The biological consequences of these transcriptional changes, including their potential relationship to phosphoinositide-dependent inflammatory signaling, require further investigation.SLC2A14 (GLUT14) should be interpreted with particular caution. Although GLUT14 is closely related to GLUT3 and has been proposed to function as a facilitative hexose transporter, its biological role is best characterized in reproductive tissues and remains poorly understood in other cell types [123]. Its specific function in human gingival fibroblasts has not been established. Therefore, the observed regulation of SLC2A14 provides evidence of an additional HU-308-associated transcriptional response within the SLC2 family but does not establish a functional role for this transporter in inflammatory metabolism.

Taken together, the changes observed across these transporter genes are consistent with the broader concept that inflammatory signaling is accompanied by alterations in cellular substrate-handling pathways. Their modulation by HU-308 may therefore represent one component of the broader transcriptional response associated with attenuation of IL-1β-induced inflammatory activation. Importantly, however, the present study measured transporter transcript abundance rather than transporter protein expression, substrate uptake, or metabolic activity. Consequently, it cannot be determined whether the observed transcriptional changes correspond to alterations in glucose or fructose uptake, myo-inositol transport, glycolytic activity, mitochondrial respiration, or overall cellular metabolic state.

Future studies integrating transporter protein expression with functional metabolic measurements, including substrate uptake, lactate production, extracellular acidification rate (ECAR), and oxygen consumption rate (OCR), will be important for determining whether the transcriptional changes identified here translate into functional metabolic adaptations. Thus, the present findings provide a transcriptional basis for investigating a potential relationship between HU-308-sensitive signaling, inflammatory activation, and cellular metabolism, while functional studies will be required to establish whether HU-308 directly modifies metabolic reprogramming in gingival fibroblasts [111,112,113,114,115,116,117,118,119,120,121,122,123,124].

### 3.5. HU-308 Modulates GPR-Associated Transcriptional Responses During Inflammatory Stimulation

The present study further demonstrates that inflammatory activation extends beyond modulation of cytokines, extracellular matrix remodeling, nitric oxide signaling, and cellular metabolism to include widespread alterations in GPR-associated transcriptional programs. GPRs constitute the largest family of membrane receptors in mammals and regulate diverse biological processes, including inflammatory signaling, chemotaxis, cellular metabolism, pH sensing, proliferation, differentiation, and tissue repair. Given the broad involvement of GPRs in these inflammation-related cellular processes, selected GPR-associated transcripts were examined to explore whether inflammatory stimulation and HU-308 treatment were accompanied by changes in GPR-related gene expression in HGFs. Although CB2 itself belongs to the GPR superfamily, comparatively little is known regarding how inflammatory stimulation influences the expression of other GPR family members in periodontal fibroblasts.

IL-1β stimulation significantly increased the expression of multiple GPR-related transcripts, including GPR160, GPR180, GPR157, GPR61, GPR173, GPR155, GPR107, and GPR75, while simultaneously suppressing GPR78 and GPR4. These changes suggest that inflammatory stimulation influences the transcriptional profile of multiple GPRs in gingival fibroblasts. Given the diverse roles of GPCRs in sensing extracellular signals, such transcriptional regulation may represent part of the broader cellular adaptation to an inflammatory microenvironment. However, changes in GPR transcript abundance do not necessarily indicate corresponding changes in receptor protein expression, receptor activation, or downstream signaling activity.

HU-308 attenuated or partially normalized several of the IL-1β-associated changes in GPR transcript expression, suggesting that its transcriptional effects during inflammatory stimulation extend to genes encoding other GPRs. Given the interconnected nature of GPR signaling and the sharing of downstream signaling components among different receptor systems, these observations raise the possibility that GPR-associated transcription represents an additional component of the cellular response to HU-308. However, the present transcriptomic findings do not establish direct interactions or functional crosstalk between CB2 and the other GPRs examined. Further receptor-specific and functional studies will be required to determine the biological significance of these transcriptional associations.

Among the receptors examined, GPR4 may be of particular interest because it functions as a proton-sensing GPCR that becomes activated under acidic extracellular conditions. Tissue acidosis is a well-recognized feature of chronic inflammation resulting from increased glycolytic metabolism, immune cell activation, and impaired tissue perfusion. Downregulation of GPR4 by IL-1β therefore suggests that inflammatory activation may alter fibroblast responsiveness to local pH changes, whereas restoration of GPR4 expression following HU-308 treatment may partially preserve acid-sensing mechanisms involved in tissue homeostasis.

Similarly, GPR75 has attracted increasing attention because it has been linked to CXCL5 (RANTES)-associated signaling and inflammatory cell recruitment. The marked induction of GPR75 observed following IL-1β stimulation, together with its attenuation by HU-308, is particularly interesting because the present study also demonstrated parallel regulation of CXCL5, suggesting coordinated remodeling of both chemokine production and chemokine-associated receptor signaling. Although the precise functional relationship between these pathways in gingival fibroblasts remains to be established, the findings raise the possibility that CB2 activation simultaneously modulates inflammatory ligand production and receptor responsiveness.

The biological functions of several additional receptors identified in the present study, including GPR160, GPR180, GPR157, GPR155, GPR107, and GPR78, remain incompletely characterized. Consequently, the present findings should be interpreted as evidence of coordinated transcriptional remodeling of GPCR-associated signaling networks rather than direct evidence of altered receptor function. Future studies should determine whether these transcriptional changes translate into alterations in receptor protein expression, ligand responsiveness, intracellular calcium signaling, cAMP production, MAPK activation, β-arrestin recruitment, chemotaxis, and fibroblast inflammatory behavior.

Collectively, these findings expand the current understanding of CB2 biology by suggesting that its anti-inflammatory effects extend beyond suppression of classical inflammatory mediators to include coordinated remodeling of the broader GPCR signaling landscape during periodontal inflammation.

### 3.6. Integrated Patterns Across Selected IL-1β-Responsive Transcriptional Domains

A major strength of the present study is the integrated analysis of multiple biologically interconnected pathways within the same experimental model. Previous investigations examining the role of CB2 signaling in inflammatory diseases have primarily focused on individual cytokines, isolated signaling cascades, or single functional outcomes. While these studies have established the anti-inflammatory properties of CB2 activation, they provide only a limited understanding of how CB2 signaling influences the complex molecular networks that collectively drive periodontal inflammation. By simultaneously evaluating inflammatory cytokines, chemokines, extracellular matrix-associated genes, nitric oxide signaling components, metabolic transporters, and GPCR-associated transcripts, the present study provides a more comprehensive view of the transcriptional responses regulated by CB2 activation in human gingival fibroblasts.

One of the most notable observations was that HU-308 did not simply suppress inflammatory gene expression indiscriminately. Instead, HU-308 treatment produced differential regulations across individual pathways. Several transcripts, including IL1B, IL11, IL23A, IL24, IL10, IL15, COL1A1, COL3A1, NOS3, and SLC2A5, were modulated to expression levels comparable with untreated controls, suggesting effective normalization of these inflammatory responses. In contrast, other genes, including IL6, IL33, MMP1, MMP3, MMP10, COL11A1, COL12A1, SLC2A1, SLC2A3, SLC2A13, and SLC2A14, demonstrated only partial recovery, whereas PRKG1 and PDE5A exhibited expression patterns extending beyond basal levels. These findings indicate that CB2 signaling selectively regulates downstream transcriptional programs rather than functioning as a simple molecular switch that uniformly suppresses inflammation.

The coordinated regulation observed across these pathways suggests that inflammatory signaling in gingival fibroblasts operates as an interconnected biological network. IL-1β-induced cytokine production promotes chemokine expression, resulting in recruitment of inflammatory cells that further amplify cytokine release and stimulate matrix metalloproteinase production. Enhanced proteolytic activity subsequently drives degradation of extracellular matrix components while suppressing collagen synthesis, thereby contributing to connective tissue destruction. Simultaneously, inflammatory activation disrupts nitric oxide signaling and promotes metabolic reprogramming, changes that further reinforce inflammatory activation and cellular dysfunction. HU-308 attenuated transcriptional changes throughout each stage of this interconnected network, suggesting that HU-308 treatment regulates multiple levels of the inflammatory response simultaneously.

This systems-level regulation has important biological implications. Chronic inflammatory diseases, including periodontitis, are increasingly recognized as disorders involving complex interactions among inflammatory, structural, metabolic, and redox signaling pathways rather than isolated alterations in individual cytokines or signaling molecules. Therapeutic strategies targeting a single inflammatory mediator have often produced limited clinical benefit because redundant signaling pathways compensate for the inhibition of individual molecules. In contrast, modulation of an upstream regulatory pathway that can coordinate multiple downstream biological processes may provide a more effective approach to limiting chronic inflammation while preserving normal tissue homeostasis.

Although the present study was performed in an in vitro model of IL-1β-stimulated human gingival fibroblasts, the integrated pathway model generated from these data provides a conceptual framework for future mechanistic and translational studies. Validation of these findings in primary periodontal tissues, animal models of periodontitis, and clinical samples will be important to determine whether the coordinated transcriptional responses observed here translate into improved tissue preservation and clinical outcomes. Furthermore, investigation of the downstream signaling mechanisms linking CB2 modulation to these interconnected pathways, including NF-κB, MAPK, PI3K/Akt, and β-arrestin-dependent signaling, may further clarify the molecular basis of the broad regulatory effects observed in the present study.

**Figure 8:**
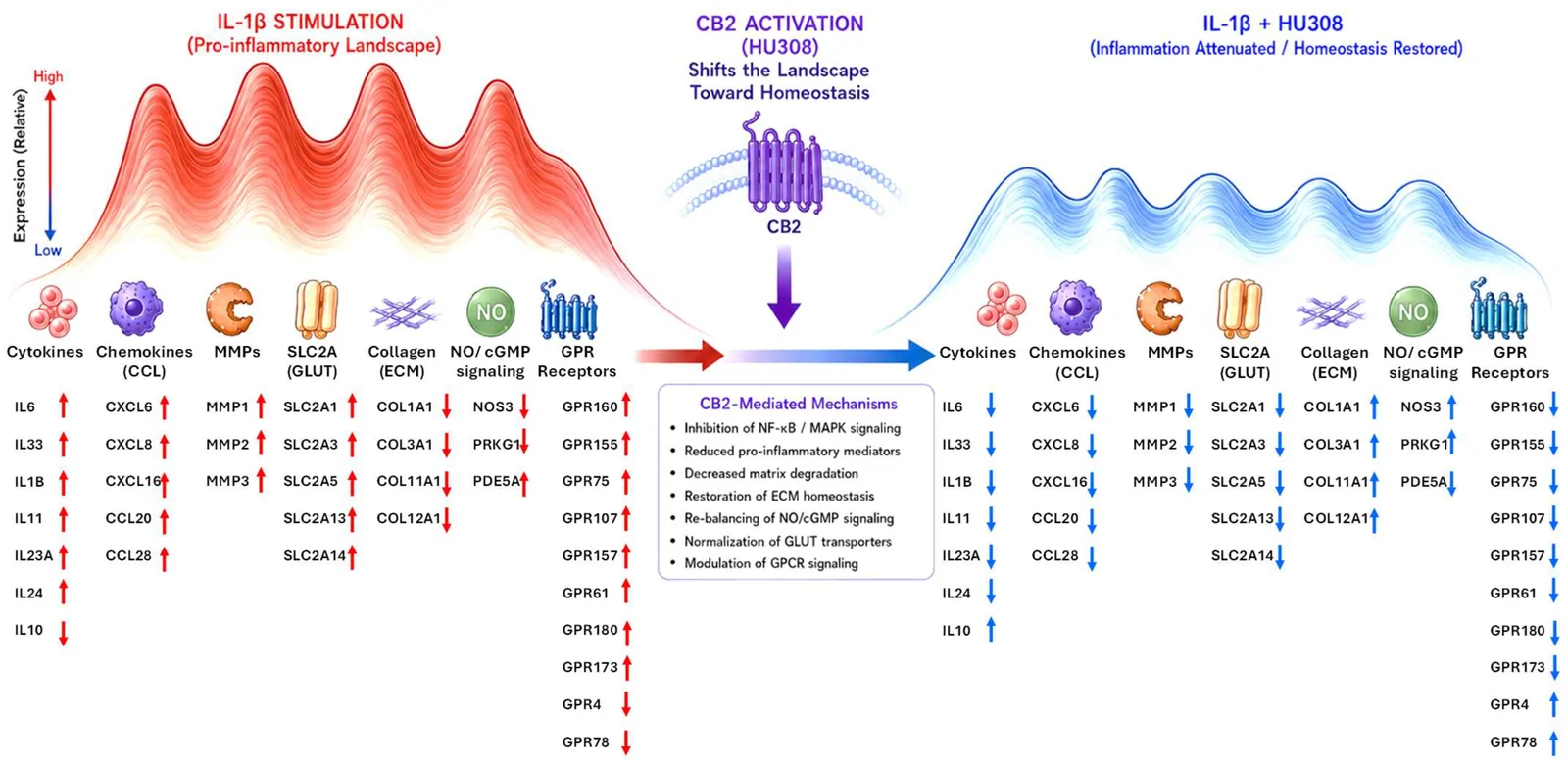
**Schematic summary of the coordinated anti-inflammatory effects of CB2 receptor activation in IL-1β-stimulated human gingival fibroblasts.** IL-1β stimulation establishes a pro-inflammatory transcriptional landscape characterized by coordinated upregulation of inflammatory cytokines, chemokines, matrix metalloproteinases (MMPs), glucose transporter (SLC2/GLUT) genes, and multiple G protein-coupled receptor (GPCR)-related transcripts, together with suppression of collagen-associated genes and nitric oxide (NO)/cGMP signaling components. Collectively, these changes promote inflammatory amplification, extracellular matrix degradation, metabolic reprogramming, impaired tissue repair, and disruption of periodontal homeostasis (**left panel**). Pharmacological modulation of the cannabinoid receptor type 2 (CB2) with the selective agonist HU-308 shifts this inflammatory landscape toward a more homeostatic phenotype (center panel). HU-308 broadly attenuates IL-1β-induced inflammatory responses by reducing the expression of pro-inflammatory cytokines, chemokines, MMPs, glucose transporter-related genes, and several GPR-associated transcripts, while restoring collagen gene expression, NO/cGMP signaling, and selected receptor-associated pathways (**right panel**). Upward and downward arrows beneath each biological domain summarize the direction of transcript changes for individual genes following IL-1β stimulation (red arrows) and subsequent HU-308 treatment (blue arrows). Overall, the integrated pathway analysis demonstrates that CB2 modulation functions as an upstream regulator of multiple interconnected biological networks, including inflammatory signaling, extracellular matrix remodeling, redox homeostasis, metabolic adaptation, and GPR-associated signaling, rather than acting on a single molecular pathway, thereby supporting a systems-level mechanism through which CB2 modulation may preserve periodontal tissue homeostasis during inflammation. This figure represents a conceptual summary of selected transcriptional patterns and does not constitute a statistically derived gene-network, pathway-enrichment, or genome-wide differential-expression analysis.

Collectively, the present findings support a proposed model in which HU-308-sensitive signaling is associated with coordinated modulation of multiple IL-1β-responsive biological pathways. Although this pattern is consistent with the hypothesis that CB2 may occupy a broader regulatory position within these networks, the present pharmacological data do not establish CB2 as an upstream regulator or exclude CB2-independent effects. The integrated pathway model proposed here suggests that coordinated modulation of inflammatory signaling, immune-cell recruitment, extracellular matrix turnover, nitric oxide signaling, and metabolic adaptation may collectively underlie the tissue-protective effects of HU-308 during periodontal inflammation (Figure 8).

### 3.7. Study Limitations

Several limitations of the present study should be acknowledged. First, the study was performed using an in vitro model of IL-1β-stimulated human gingival fibroblasts, which, although widely used to investigate periodontal inflammation, cannot fully reproduce the complex cellular interactions that occur within periodontal tissues. In vivo, gingival fibroblasts interact dynamically with epithelial cells, endothelial cells, immune cells, osteoblasts, osteoclasts, and the oral microbiota, all of which contribute to disease progression and resolution. Consequently, the transcriptional responses observed in isolated fibroblasts should be interpreted within the context of this simplified experimental model.

In addition, IL-1β exposure represents a defined experimental inflammatory stimulus and should not be considered equivalent to periodontitis. Periodontitis is initiated and sustained within a complex microenvironment involving dysbiotic microbial communities and interactions among epithelial, stromal, immune, vascular, and bone-remodeling compartments. These components are not reproduced in the present experimental model. Accordingly, the findings should be interpreted specifically as transcriptional responses of HGFs to IL-1β stimulation and HU-308 treatment rather than as direct evidence of molecular changes occurring in periodontitis in vivo.

Second, the present study focused primarily on transcriptomic alterations. Although changes in gene expression provide important insight into the biological pathways regulated by CB2 activation, mRNA abundance does not necessarily correlate with protein expression or functional activity. Future studies should therefore validate these findings at the protein level using approaches such as Western blotting, ELISA, immunofluorescence, or proteomic analyses and determine whether the observed transcriptional changes translate into functional alterations in cytokine secretion, extracellular matrix remodeling, nitric oxide production, and cellular metabolism.

Third, although the present findings suggest coordinated regulation of multiple inflammatory pathways, the downstream signaling mechanisms responsible for these effects were not directly investigated. CB2 receptors signal through several intracellular pathways, including inhibition of cAMP production, modulation of PI3K/Akt, ERK1/2, p38 MAPK, JNK, NF-κB, and β-arrestin-dependent signaling. Defining which of these pathways mediate the broad transcriptional responses observed following HU-308 treatment will require additional mechanistic studies using CB2 antagonism or CNR2 knockdown. Functional assays should be performed to evaluate MMP activity, collagen deposition, nitric oxide/cGMP signaling, ROS generation, and glucose uptake.

Finally, the current study evaluated gene expression at a single experimental time point following IL-1β stimulation. Because inflammatory signaling is highly dynamic, future investigations examining the temporal regulation of CB2-responsive genes, together with dose-response studies and validation in animal models and clinical periodontal tissues, will provide a more comprehensive understanding of the therapeutic potential of CB2 activation during periodontal disease progression.

Independent RT-qPCR validation of the selected microarray-derived transcriptional findings was not performed in a separate biological cohort. This represents an important limitation, particularly given the small number of biological replicates, the targeted selection of transcripts for downstream analysis, and the absence of across-transcript multiplicity correction. Accordingly, the reported gene-level associations should be considered exploratory and hypothesis-generating until reproduced in independent biological samples. Future validation should include CNR2 together with representative transcripts from the inflammatory, extracellular-matrix, and NO/cGMP-associated domains using an independent biological cohort.

### 3.8. Clinical Implications and Future Directions

Current host-modulatory therapies for periodontitis primarily target individual inflammatory mediators or broadly suppress inflammation, approaches that often provide limited efficacy because of redundancy within inflammatory signaling networks. The present findings demonstrate that HU-308 treatment is associated with modulation of multiple IL-1β-responsive transcriptional domains in HGFs, including inflammatory mediators, extracellular matrix-associated genes, NO/cGMP-related genes, transporter-associated genes, and GPCR-related transcripts. The involvement of several transcriptional domains suggests that the response to HU-308 under inflammatory conditions is not restricted to a single group of genes. However, these findings were obtained in an IL-1β-stimulated HGF monoculture and should not be interpreted as evidence that HU-308 regulates the corresponding functional pathways in periodontal tissues or provides therapeutic benefit in periodontitis.

From a translational perspective, selective CB2 agonists possess several attractive characteristics. Because CB2 receptors are expressed predominantly within immune and peripheral tissues, selective agonists such as HU-308 are expected to exert anti-inflammatory effects without the psychoactive adverse effects associated with CB1 receptor activation. Selective CB2 agonists remain of interest as potential host-modulatory agents because CB2-associated signaling has been implicated in the regulation of inflammatory responses. The present findings provide additional rationale for investigating HU-308 in more physiologically relevant periodontal models but are not sufficient to establish its therapeutic efficacy. Studies incorporating receptor-specific approaches, multicellular systems, microbial components, animal models of periodontitis, and ultimately human periodontal tissues will be necessary before the translational potential of HU-308 can be determined.

The broad biological effects observed in the present study also suggest potential applications beyond periodontal disease. Chronic inflammatory disorders, including peri-implantitis, rheumatoid arthritis, inflammatory bowel disease, diabetes, and cardiovascular diseases, share common pathological mechanisms involving dysregulated cytokine production, oxidative stress, extracellular matrix remodeling, and metabolic dysfunction. Therefore, further investigation of CB2 signaling across these interconnected inflammatory conditions may identify common therapeutic mechanisms with broader clinical relevance.

Future studies should validate these findings using three-dimensional periodontal tissue models, co-culture systems incorporating immune cells, and animal models of periodontitis. In addition, comprehensive transcriptomic, proteomic, metabolomic, and single-cell sequencing approaches will further define the molecular mechanisms through which HU-308 regulates periodontal homeostasis and identify biomarkers predictive of therapeutic response. Such studies will be essential for determining whether the transcriptional changes observed in the present investigation translate into clinically meaningful preservation of periodontal tissues.

Furthermore, the study relied on a single pharmacological agonist, HU-308, at one concentration. Although HU-308 is commonly used as a selective CB2 agonist, receptor selectivity under the present experimental conditions was not directly verified. The absence of a CB2 antagonist, CNR2 knockdown or knockout, and a second structurally distinct CB2 agonist prevents definitive attribution of the observed responses to CB2. The findings should therefore be regarded as pharmacological evidence supporting, but not proving, the involvement of CB2 signaling.

In addition, cell viability and cytotoxicity were not independently quantified under the experimental conditions used for transcriptomic analysis. Therefore, although the selected HU-308 concentration was based on previously employed experimental conditions, a contribution of treatment-related effects on cell health cannot be completely excluded. Future studies should incorporate direct viability assessment together with dose-response and time-course analyses.

“Although multiple candidate genes were analyzed, the study employed a hypothesis-driven targeted gene panel rather than an exploratory genome-wide approach. Consequently, statistical analyses were performed using one-way ANOVA with Tukey’s post hoc test without applying a global false discovery rate correction across genes. While this approach is appropriate for targeted analyses, it may increase the likelihood of false-positive findings and should be considered when interpreting the results. Future studies with larger sample sizes and independent validation cohorts will be important to confirm these findings.”

The study evaluated only one concentration of IL-1β and HU-308 and one 24-h time point. Because inflammatory and cannabinoid-responsive signaling are dynamic, additional dose-response and time-course studies are required to determine the onset, magnitude, and persistence of the observed transcriptional effects.

The experimental design did not include an HU-308-alone condition. Consequently, the present study cannot determine whether HU-308 independently alters the examined transcripts under basal conditions, nor can it formally test an IL-1β × HU-308 interaction. In addition, because a vehicle-only control was not included, the independent contribution of the HU-308 vehicle to the observed transcriptional responses cannot be excluded. Future studies incorporating HU-308-alone and vehicle-matched control groups will be required to distinguish these effects.

Because a vehicle-only condition was not included, a contribution of the vehicle to the observed transcriptional responses cannot be completely excluded.

The selected microarray-derived transcriptional findings were not independently validated by RT-qPCR in a separate biological cohort. Independent validation of representative transcripts, including CNR2 and genes from the inflammatory, extracellular-matrix, and NO/cGMP-associated domains, will be required to confirm the reproducibility of these observations.

An additional limitation is that the downstream statistical analysis was performed on selected normalized transcript values using gene-wise ANOVA with Tukey’s multiple-comparison testing, without correction for multiplicity across all examined transcripts. Consequently, the probability of false-positive gene-level associations is increased, and the reported *p* values should be interpreted as nominal and exploratory. Future studies should apply dedicated transcriptomic statistical frameworks with appropriate multiple-testing control and independent validation of representative transcripts.

An immediate priority for follow-up studies is independent validation of representative microarray-derived signals in a separate biological cohort. RT-qPCR studies should include CNR2 and representative inflammatory, extracellular-matrix, and NO/cGMP-associated transcripts. Where appropriate, transcript-level validation should also be complemented by protein-level and functional assays to determine whether the observed transcriptional associations translate into corresponding biological effects.

## 4. Materials and Methods

### 4.1. Cell Culture of Primary Human Gingival Fibroblasts (HGFs)

Primary human gingival fibroblasts (HGFs) were obtained commercially (ATCC, Manassas, VA, USA, CRL2014). Donor information is not available. Cells were cultured in fibroblast basal medium supplemented with manufacturer-recommended growth factors, 10% fetal bovine serum (FBS), and antibiotics (1% penicillin/streptomycin) (ATCC, Manassas, VA, USA), and maintained at 37 °C in a humidified incubator containing 5% CO_2_. Cells were passaged upon reaching 70–80% confluency and used between passages 3 and 6 to ensure phenotypic consistency and stability.

### 4.2. Experimental Design and Treatment Conditions

HGFs were seeded at a density of approximately 1 × 10^6^ cells per well and allowed to adhere overnight before experimental treatments. Cells were divided into three experimental groups: untreated control, IL-1β-treated, and IL-1β followed by HU-308 treatment. To induce a disease-like inflammatory phenotype, HGFs were stimulated with IL-1β at 10 ng/mL. In the treatment group, HU-308 was added at 10 μM one hour after IL-1β stimulation. This sequential treatment design was selected to initiate the inflammatory response before HU-308 exposure and thereby model an early therapeutic intervention rather than simultaneous prophylactic treatment. The one-hour interval was not intended to establish that a fully developed inflammatory phenotype had occurred before HU-308 administration. Although previous studies using related primary periodontal fibroblast models have reported tolerability of HU-308 at comparable concentrations, cell viability was not independently quantified in the present experiment. This represents a limitation and should be addressed in future studies. Cells were harvested 24 h after the initial IL-1β stimulation. All experiments were conducted using biological triplicates (*n* = 3 per group) to support reproducibility. Experiments were conducted using primary human gingival fibroblasts (HGFs) obtained from three independent human donors. Cells from each donor were included across all experimental conditions. Accordingly, *n* = 3 represents three independent biological donors.

Three-dimensional pathway diagrams were generated as schematic visualizations to summarize coordinated changes in gene expression within each biological pathway. Each node represents an individual gene, and node size was scaled according to the relative mean normalized expression observed in each experimental group. The connecting lines were included solely to illustrate relationships among genes within the same functional pathway and do not represent experimentally determined molecular interactions, correlation coefficients, or regulatory connections. These diagrams were intended as graphical summaries of the transcriptomic findings rather than formal gene co-expression network analyses.

Recombinant human IL-1β was obtained from (InvivoGen, San Diego, CA, USA), reconstituted in water according to the manufacturer’s instructions, and used at a final concentration of 10 ng/mL. HU-308, a selective CB2 receptor agonist (Tocris Bioscience/R&D Systems, Minneapolis, MN, USA, Cat. No. 3088; purity ≥ 98% by HPLC), was dissolved in dimethyl sulfoxide (DMSO) to prepare a 10 mM stock solution. Immediately before treatment, the stock solution was diluted 1:1000 in the appropriate cell culture medium to achieve a final HU-308 concentration of 10 µM. The resulting final DMSO concentration in the culture medium was 0.1% (*v*/*v*). Vehicle-control cultures received an equivalent final concentration of DMSO (0.1% *v*/*v*).

### 4.3. RNA Isolation

Human gingival fibroblasts (HGFs) were cultured in basal medium supplemented with the appropriate growth factors under the experimental conditions described above. For RNA isolation, approximately 1 × 10^6^ cells were lysed directly in the culture dish, and total RNA was isolated using the QIAcube automated platform (QIAGEN, Hilden, Germany). Following isolation, RNA samples were concentrated by ethanol precipitation. RNA concentration and purity were assessed using a NanoDrop spectrophotometer (Thermo Fisher Scientific, Wilmington, DE, USA), with an A260/A230 ratio > 1.6 used as an acceptance criterion. Qualified RNA samples were diluted to a final concentration of 100 ng/µL in DNase-free water and subsequently processed for transcriptomic analysis using Affymetrix Human Clariom S arrays (Santa Clara, CA, USA) and the Affymetrix transcription assay according to the manufacturer’s protocol.

### 4.4. RNA Quality Control

RNA concentration and purity were assessed using a NanoDrop spectrophotometer, with an A260/A230 ratio >1.6 required for downstream processing. RNA integrity was evaluated using an Agilent 2100 Bioanalyzer (Agilent Technologies, Santa Clara, CA, USA) with the Eukaryote Total RNA Nano assay, and samples with a RIN > 8 were considered acceptable for microarray analysis. Qualified RNA was diluted to 100 ng/µL in DNase-free water, and approximately 1 µg of RNA per sample was submitted to the UTHSC Molecular Resource Center.

Transcriptomic profiling was performed using Affymetrix Human Clariom S arrays with the Affymetrix WT transcription assay workflow. Following array processing, expression data were normalized using Affymetrix Expression Console software 1.4.1 (Affymetrix, Inc., Santa Clara, CA, USA). Array quality was assessed using the internal and reference control probes incorporated into the Affymetrix assay workflow before downstream analysis.

Normalized expression data were exported for statistical analysis. Gene identifiers, accession numbers, and corresponding expression values were retained, while non-annotated features were excluded. Pairwise comparisons between experimental groups were performed using Welch’s *t*-test. Descriptive statistics, including mean, variance, standard deviation, standard error, coefficient of variation, and fold change, were calculated for each comparison. To account for multiple testing, *p*-values were adjusted using the Benjamini–Hochberg false-discovery-rate procedure, with an adjusted *p*-value < 0.05 considered statistically significant.

### 4.5. Microarray Processing

A total of 1 μg of RNA per sample was submitted to the University of Tennessee Health Science Center (UTHSC) Molecular Resource Center for processing. Samples were prepared using the Affymetrix WT Transcription Assay Kit (Affymetrix, Inc., Santa Clara, CA, USA) and hybridized to Affymetrix Human Clariom S arrays, enabling transcriptome-wide profiling of more than 20,000 genes, including both coding and non-coding transcripts. The microarray data analyzed in the present study consisted exclusively of the three experimental conditions described in Section 2.2: untreated control HGFs, IL-1β-stimulated HGFs, and IL-1β-stimulated HGFs subsequently treated with HU-308. This unified microarray dataset served as the foundation for all downstream pathways and domain-specific analyses.

### 4.6. Data Normalization and Preprocessing

Raw microarray data were processed and normalized using Affymetrix Expression Console software employing the Robust Multi-array Average (RMA) normalization method. Quality control measures included assessment of internal probe performance, signal distribution consistency, and background correction. Following normalization, datasets were exported as CSV files for downstream analysis and interpretation.

### 4.7. Bioinformatic and Statistical Analysis

Following microarray processing and normalization by the UTHSC Molecular Resource Center, normalized expression data were exported as CSV files and imported into GraphPad Prism 9 (Boston, MA, USA) for downstream statistical analysis. Only annotated transcripts were retained for analysis, and non-informative probes and duplicate entries were removed.

For the selected transcripts presented in the figures, differences among the three experimental groups were assessed separately for each gene using one-way ANOVA followed by Tukey’s multiple-comparison test. Tukey’s procedure accounted for pairwise comparisons among treatment groups within each individual transcript. Data are presented as mean ± standard error of the mean (SEM), with statistical significance thresholds defined as * *p* < 0.05, ** *p* < 0.01, *** *p* < 0.001, and **** *p* < 0.0001.

No additional false-discovery-rate correction was applied across the complete set of transcripts examined. Accordingly, the reported *p* values should be considered nominal with respect to across-transcript multiplicity, and the gene-level findings are interpreted as exploratory and hypothesis-generating rather than as confirmatory genome-wide differential-expression results. Because no across-gene multiplicity correction was applied, individual *p* values should be interpreted as exploratory rather than confirmatory.

### 4.8. Targeted Domain-Based Analysis of Selected Transcripts

The present study represents a targeted secondary analysis of selected microarray-derived transcripts grouped according to predefined biological relevance. It was not designed as an unbiased genome-wide differential-expression, pathway-enrichment, or gene-network analysis.

Genes were grouped into biologically relevant domains based on functional annotation and established biological relevance, including interleukin signaling, chemokine-associated responses, extracellular matrix remodeling, nitric oxide/cGMP-associated signaling, solute carrier transporter expression, and GPCR-related transcription. These domains were evaluated to identify patterns of transcriptional response associated with IL-1β stimulation and subsequent HU-308 treatment.

GPCR-associated transcripts were examined as an exploratory functional domain within the targeted secondary analysis of the normalized microarray dataset. GPCRs participate in multiple cellular processes relevant to inflammatory fibroblast biology, including inflammatory signaling, chemotaxis, extracellular sensing, cellular metabolism, pH sensing, proliferation, differentiation, and tissue repair. Selected GPCR-related transcripts were therefore evaluated to determine whether IL-1β stimulation and subsequent HU-308 treatment were associated with changes in their transcript abundance. This analysis was not intended to represent a comprehensive GPCR screen, and inclusion of these transcripts should not be interpreted as evidence of established functional roles in HGFs or direct interactions with CB2.

## 5. Conclusions

In summary, the present study demonstrates that activation of the CB2 receptor by the selective agonist HU-308 exerts broad transcriptional effects in IL-1β-stimulated human gingival fibroblasts. Rather than regulating isolated inflammatory mediators, HU-308 coordinated the modulation of multiple biologically interconnected pathways involved in periodontal inflammation, including cytokine and chemokine signaling, extracellular matrix remodeling, nitric oxide/cGMP signaling, metabolic transporter expression, and GPCR-associated transcriptional networks.

These coordinated transcriptional changes indicate that HU-308 treatment in the presence of IL-1β is associated with reduced inflammatory amplification and with changes in extracellular matrix, nitric oxide/cGMP, metabolic, and GPCR-related transcripts. The findings support a hypothesis in which CB2 signaling may contribute to the coordinated regulation of multiple IL-1β-responsive pathways rather than acting through a single downstream mediator. However, because the study used one agonist at one concentration and did not include CB2 antagonism, CNR2 knockdown, or a second structurally distinct agonist, receptor-specific causality cannot be concluded.

Although the present findings provide a rationale for further investigation of HU-308 and CB2-associated signaling in the context of periodontal inflammation, their translational significance remains to be established. The IL-1β-stimulated HGF monoculture used in this study represents a reductionist inflammatory model and does not reproduce the microbial, immune, epithelial, vascular, and bone-remodeling interactions characteristic of periodontitis. Therefore, the present results should be interpreted as hypothesis-generating transcriptional observations rather than evidence of therapeutic efficacy. Receptor-specific studies, functional validation, multicellular experimental systems, animal models of periodontitis, and human periodontal tissues will be necessary to determine whether these transcriptional responses translate into biologically meaningful or therapeutically relevant effects.

## Data Availability

The original data presented in the study are openly available in https://doi.org/10.5281/zenodo.22371906.
